# Divergence in Morris Water Maze-Based Cognitive Performance under Chronic Stress Is Associated with the Hippocampal Whole Transcriptomic Modification in Mice

**DOI:** 10.3389/fnmol.2017.00275

**Published:** 2017-08-30

**Authors:** Seung H. Jung, Milene L. Brownlow, Matteo Pellegrini, Ryan Jankord

**Affiliations:** ^1^Applied Neuroscience, Warfighter Interface Division, 711th Human Performance Wing, Air Force Research Laboratory Wright-Patterson AFB, OH, United States; ^2^Research Associateship Program, National Research Council, National Academies of Science Washington, DC, United States; ^3^Department of Molecular, Cell, and Developmental Biology, University of California, Los Angeles Los Angeles, CA, United States

**Keywords:** spatial learning, contextual memory, chronic variable stress, whole transcriptome, upstream regulator analysis, WGCNA (Weighted Gene Co-expression Network Analyses), PANTHER (Protein Analysis Through Evolutionary Relationships), alternative splicing

## Abstract

Individual susceptibility determines the magnitude of stress effects on cognitive function. The hippocampus, a brain region of memory consolidation, is vulnerable to stressful environments, and the impact of stress on hippocampus may determine individual variability in cognitive performance. Therefore, the purpose of this study was to define the relationship between the divergence in spatial memory performance under chronically unpredictable stress and an associated transcriptomic alternation in hippocampus, the brain region of spatial memory consolidation. Multiple strains of BXD (B6 × D2) recombinant inbred mice went through a 4-week chronic variable stress (CVS) paradigm, and the Morris water maze (MWM) test was conducted during the last week of CVS to assess hippocampal-dependent spatial memory performance and grouped animals into low and high performing groups based on the cognitive performance. Using hippocampal whole transcriptome RNA-sequencing data, differential expression, PANTHER analysis, WGCNA, Ingenuity's upstream regulator analysis in the Ingenuity Pathway Analysis® and phenotype association analysis were conducted. Our data identified multiple genes and pathways that were significantly associated with chronic stress-associated cognitive modification and the divergence in hippocampal dependent memory performance under chronic stress. Biological pathways associated with memory performance following chronic stress included metabolism, neurotransmitter and receptor regulation, immune response and cellular process. The Ingenuity's upstream regulator analysis identified 247 upstream transcriptional regulators from 16 different molecule types. Transcripts predictive of cognitive performance under high stress included genes that are associated with a high occurrence of Alzheimer's and cognitive impairments (e.g., Ncl, Eno1, Scn9a, Slc19a3, Ncstn, Fos, Eif4h, Copa, etc.). Our results show that the variable effects of chronic stress on the hippocampal transcriptome are related to the ability to complete the MWM task and that the modulations of specific pathways are indicative of hippocampal dependent memory performance. Thus, the divergence in spatial memory performance following chronic stress is related to the unique pattern of gene expression within the hippocampus.

## Introduction

The incidence rate of neurological diseases and disorders, especially related to cognition and memory performance, has dramatically increased in recent years (UN, 2015). Chronic stress is recognized as one of the main contributors to these disorders (Heller et al., 2014; Hodes et al., 2015; Mcewen et al., 2015), and individuals' stress levels are reported to have increased in the past few years (American Psychological Association, 2015, 2016). Evidence from human and animal-based studies suggest that chronic stress contributes heavily to neurological diseases/disorders (Heller et al., 2014; Gelisse et al., 2015; Hodes et al., 2015; Mcewen et al., 2015). In particular, chronic stress has been linked to cognitive impairment (Johnson, 2015; Rickenbach et al., 2015) and Alzheimer's disease (Cuadrado-Tejedor et al., 2012; Cuadrado-Tejedor and Garcia-Osta, 2016), and may, therefore, limit the quality of life in susceptible individuals (Kim and Diamond, 2002). The underlying mechanisms by which chronic stress impairs an individual's cognitive performance remains an important area of investigation.

The hippocampus is a critical brain region for memory consolidation. Unfortunately, this brain region has been shown to be affected by a variety of stressful environments (Kim and Diamond, 2002; Mcewen et al., 2015; Stankiewicz et al., 2015). Stress has been shown to disrupt hippocampal cellular function and homeostasis, resulting in hippocampal dependent memory impairment: declarative/explicit memory in humans and spatial/contextual memory in rodents (Eichenbaum, 2000; Scoville and Milner, 2000; Kim and Diamond, 2002). Stress-induced modifications in hippocampal structures and memory function occur through complex biomolecular cascades (Kim and Diamond, 2002). A recent study that analyzed hippocampal gene profiles from psychologically stressed mice reported that prolonged exposure to stress (up to 2 weeks) modified hippocampal gene regulation, providing further evidence that stress affects the neurobiological processes within the hippocampus (Stankiewicz et al., 2015). Although chronic stress is generally known to induce adverse effects on brain and brain performance, the destructive effects of stress on hippocampal structures and memory function can vary widely across individuals. This variance in stress effects on individual performance is also observed in rodents, where a recent publication (Shea et al., 2015) showed a wide variance in spatial learning performance in mice under prolonged stressful conditions.

It has been shown that genetic, biological and environmental factors determine the stress-associated vulnerability to cognitive function (Mcewen and Gianaros, 2010). A better understanding of the underlying genetic and biological processes by which some individuals, but not others, are susceptible to stress-induced effects on hippocampal dependent memory performance will aid the development of therapeutic treatments to reduce, prevent or, reverse the detrimental effects of chronic stress on cognition. In order to address this knowledge gap, our group analyzed whole hippocampal transcriptomes from mice that completed the MWM test following chronic stress exposure. We hypothesized that the divergence in spatial memory abilities (low vs. high performing groups under chronic stress) would be associated with unique gene expression profiles. We utilized the PANTHER database and weighted gene coexpression network analysis to identify the biological pathways that were most highly related to hippocampal dependent cognitive performance under chronic stress. The pathways identified in this study will be informative for furthering our understanding of the relationship between stress susceptibility and cognitive function detected by MWM test.

## Materials and methods

### Animals

**Figure 1:**
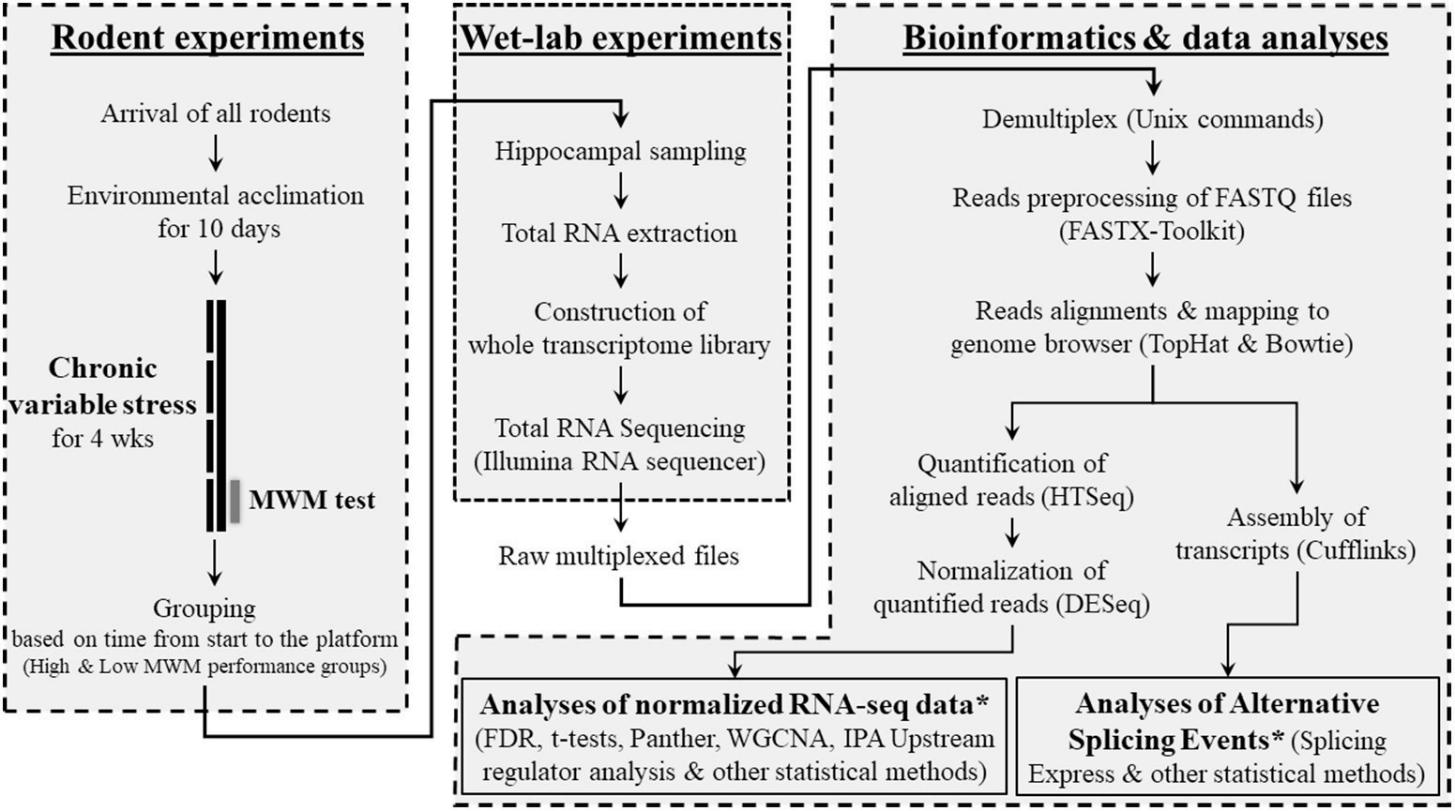
Overview of research design. This research was composed of 3 sub-research categories as shown as boxes with dashed lines. In the category of bioinformatics and data analyses, bioinformatics programs/software and data analyzing methods that used for each step were provided in parentheses. ^*^See Supplementary Figure S1 for detailed information about the data analysis methods.

**Figure 2:**
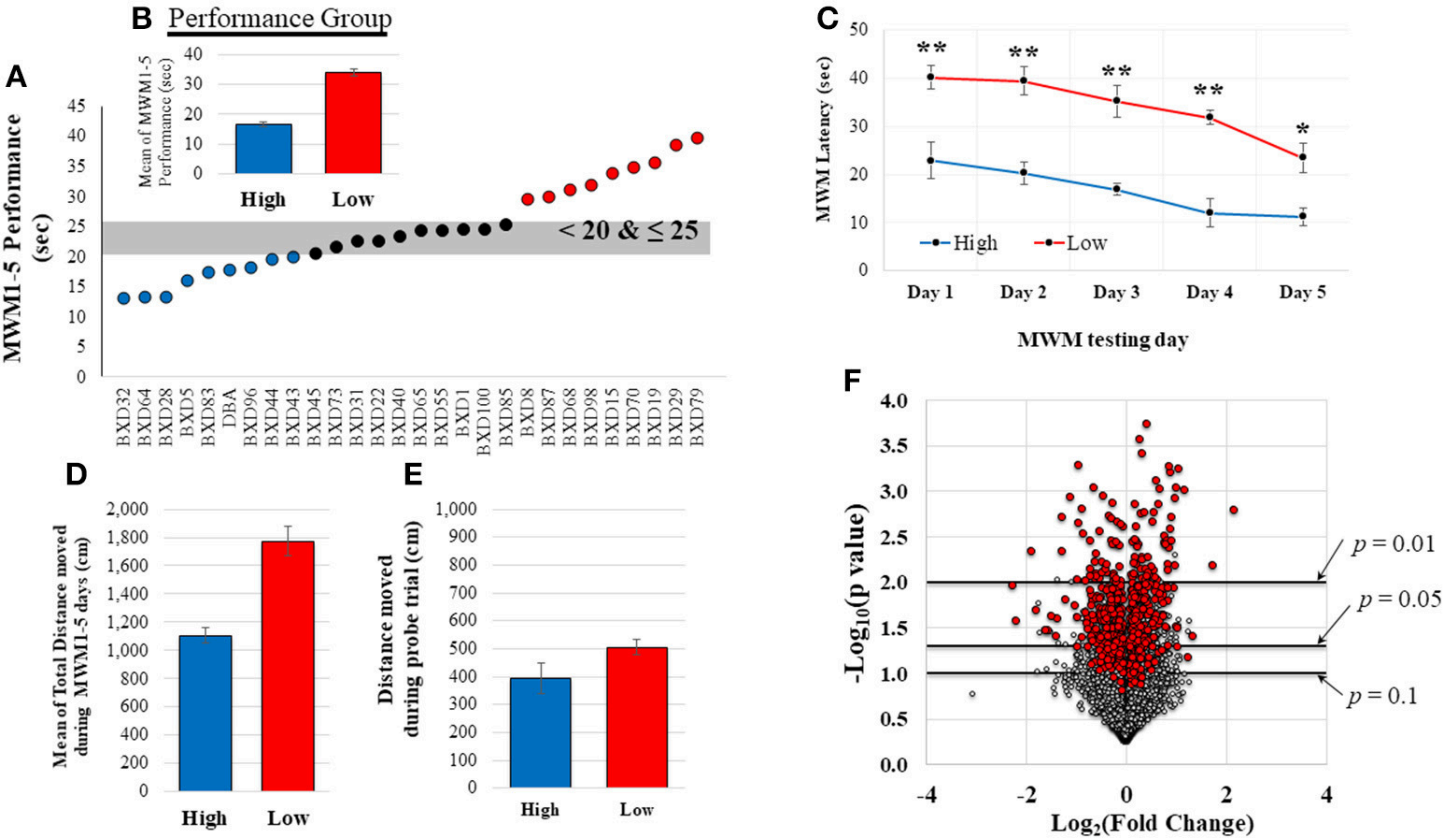
MWM performance data and distribution of normalized gene expression. **(A)** shows the range of MWM performance of these 28 mice. Based on this distribution, mice were grouped in to high (good cognitive performer, shorter latency to platform) or low (poor cognitive performer, longer latency to platform) MWM performance groups (*n* = 9/group). A significant difference between the high and low MWM performance groups was detected **(B**, *p* < 0.0001). Two-way repeated measure ANOVA also resulted in significant group and days difference **(C**, *p* < 0.001 for group and days. *post hoc*-tests showed significant group difference in each day (^**^p < 0.001 and ^*^p = 0.002). The average of total distance moved during the 5 MWM testing days was also significantly different between the groups **(D**, *p* < 0.0001). No statistical group difference was detected from the total distance moved during the probe trial (**E**, on the 6th MWM testing days, *p* = 0.84). The volcano plot **(F)** shows a distribution of fold changes (log_2_FC) and −log_10_(*p*-values) for normalized-and-filtered genes (16,565 genes). Genes (*n* = 521) with |r| ≥ 0.5 were represented as red-colored dots.

Twenty-eight male mice (9 weeks old) of 28 different strains were purchased from Jackson Laboratory (Bar Harbor, ME, USA). Animals included in this study represent only a single cohort of mice that were randomly selected from a larger behavioral genetics study (Carhuatanta et al., 2014; Shea et al., 2015) using BXD recombinant inbred strains (For all strain information, please see **Figure 2A**), derived by crossing C57BL/6K (B6) and DBA/2J (D2), that have been significantly used to study gene-phenotype interactions. All animals included in this cohort arrived to our facility at the same time and completed the experimental procedures, including stressors, behavioral testing and sample collections, on the same day. All mice were singly housed with *ad libitum* access to food and water and allowed to acclimate to the Wright-Paterson Air Force Base (WPAFB) animal facility for 10 days. Ambient housing conditions were controlled for temperature (18–24°C), humidity (30–70%), and dark/light cycle (12:12; 06:00 on). All experiments were performed during the light phase. After completing the behavioral tests, mice were euthanized via rapid decapitation and whole brains were quickly frozen and stored at −80°C until further processing. All rodent handling and procedures were performed in accordance with the National Institute of Health standards and the Guide for the Care and Use of Laboratory Animals (National Research Council), and the protocol was approved by the WPAFB Institutional Animal Care and Use Committee. An overall research outline is shown in Figure 1.

### Chronic stress paradigm

Only stress groups were selected for this study because the purpose of this study was to investigate how cognitive performance under chronically unpredictable stress environments were various and how this variety was related to transcriptomic modification in the hippocampus. The chronic variable stress (CVS) paradigm was completed over 4 consecutive weeks as described previously (Schmidt et al., 2008; Carhuatanta et al., 2014). Briefly, mice were randomly exposed to twice daily stressors (morning and afternoon) for 5 days/week. Stressors included cold exposure (15 min at 4°C), hypoxia (30 min at 8–12% O_2_), constant motion (60 min at 100 rpm), novel overnight housing, restraint (60 min) and open field (30 min in a 19″ × 10.5″ × 8″ cage) exposure. To limit predictability and habituation, stressors were presented in a randomized fashion to the mice.

### Morris water maze task

All mice underwent Morris water maze (MWM) testing during the last week as described previously (Morris, 1984; Vorhees and Williams, 2006) to examine spatial memory acquisition. Briefly, a round basin (90 cm diameter) was used for MWM task and filled with water (42 cm of depth), mixed with nontoxic white paint until a clear platform became invisible (the range of water temperature: 19–24°C). The platform was located in the southwest quadrant submerged approximately 1 cm below the water surface and all visual distal clues were provided on the walls. Rodents were given 5 training days of 4 trials per a day using a random starting location. If rodents did not reach the platform within 60 s they were gently guided to the platform. The EthoVision video tracking software (version XT7.0.418, Noldus Information Technology, Leesburg, VT) was used to record mouse swim path, position, speed, and latency to platform. For this study, the mean of MWM performance (latency time to locate platform) during the 5 days was used to group the mice (Figure 2A) into high (better cognitive performers, shorter latency to platform time: < 20 s; *n* = 9) and low (worse cognitive performers, longer latency to platform time: >25 s; *n* = 9) MWM performance groups. Probe trial, for which the platform was removed, was also given to the animals on the 6th MWM testing day. Animals that displayed average MWM performance (latency to platform time: 20–25 s; *n* = 10) were excluded from the comparative analysis between high and low performing groups. The total distance moved during the MWM testing days and the probe trial was also recorded.

### RNA sequencing (illumina RNA-seq analysis)

As soon as behavioral tests were completed (on the last day of 4 consecutive weeks for CVS), animals were immediately euthanized in about 10 min on the same day and, then, tissues were dissected and collected on ice and frozen quickly. Total RNA from hippocampus of the rodents were utilized for building library constructions and, then, running the Ilumina whole transcriptome RNA sequencer. The frozen brain was placed in the RNAlater®-ICE Frozen Tissue Transition Solution (Thermo Fisher Scientific, Waltham, MA) at −20°C for overnight and dissected hippocampus, and total RNA was extracted from the whole hippocampus using the RNeasy Mini Kit (Qiagen, Valencia, CA) according to instructions provided in the kit. RNA samples were shipped overnight to the UCLA Biosequencing core where the Illumina RNA sequencer was used for whole transcriptome RNA-sequencing. Samples were multiplexed, tagged with standard Illumina tags and sequenced using Illumina next-gen RNA-Sequencing by trained technicians.

### Bioinformatic analyses

All multiplexed data from the Illumina RNA Sequencer were downloaded and fastQ files of each sample were demultiplexed and merged into one fastQ file. All sequencing adapters were removed by running the FASTX-Toolkit, FASTQ clipper, and all reads were, then, trimmed by using another FASTX-Toolkit FASTQ trimmer (http://hannonlab.cshl.edu/fastx_toolkit/) if needed. The FastQC application (http://www.bioinformatics.babraham.ac.uk/projects/fastqc/) was used to check quality control on FastQ files. All reads were aligned to UCSC genome browser (Mus musculus NCBIM37) by using TopHat (version 2.0.6) (Trapnell et al., 2012). The aligned reads (mean: 27.5 M reads) kept by TopHat running were quantified by HTSeq (version 0.5.3p9) (Anders et al., 2015). The DESeq package (Anders and Huber, 2010) in R was used to normalize read counts. Genes that had no reads across all samples were discarded by the DESeq analysis. The PANTHER (Protein ANalysis THrough Evolutionary Relationships) Classification System was used to classify genes in addition to use of the weighted gene coexpression network analyses (WGCNA) as described below.

PANTHER Classification System (http://PANTHERdb.org) (Thomas et al., 2003) was used to classify genes that were significantly expressed detected by the DESeq analysis and correlation analysis. Lists of Ensembl gene IDs were entered into the PANTHER database to classify genes into functional groups, providing information about their biological function and pathways. The PANTHER statistical over- and under-representation tests were carried out with log_2_ fold change (log_2_FC) values and available Entrez Gene IDs to investigate whether identified biological processes were statistically over- or under-expressed.

Normalized gene expression values from DESeq data were used to construct signed coexpression networks using the WGCNA package in R (Langfelder and Horvath, 2008). After the data cleaning step, the step-by-step network construction and module detection was carried out. The adjacency was calculated with the softPower of 10 (the scale-free topology fit index SFT *R*^2^ = 0.815). The corresponding dissimilarity was calculated from topological overlap matrix (TOM) that was transformed from the adjacency, and then hierarchical clustering and dynamic tress cut methods were carried out to identify the modules with the recommended minimum module size of 30. The Dynamic Tree Cut method identified similar modules, their eigengenes were calculated, clustered based on their correlation and, then, merged modules if their correlation was > 0.75 (from 12 dynamic modules to 10 merged modules). An association of genes in each module with the traits (individual rodents' means of MWM performance during 5 days) was quantified by defining Gene Significance (GS), and a quantitative measure of module membership that is a correlation of the module eigengene and the gene expression profile was calculated. The WGCNA method identified modules with a statistically significant correlation between means of MWM performance and genes. The eigengene dendrogram function of the WGCNA was used to identify groups of correlated eigengenes, discovering mutual correlations between modules and the trait. Significantly correlated modules were used to perform GO enrichment analysis and identified the 20 best terms for each module. The top 30 genes from the highest module (magenta) were visualized with VisANT 5.0 (Hu et al., 2008) for gene connections with the thresholds of 0.02 and the result figure was shown in **Figure 4D**.

The Ingenuity's upstream regulator analysis in the Ingenuity Pathway Analysis (IPA, Ingenuity Systems, and www.ingenuity.com) was used to identify the upstream transcriptional regulators. Upstream regulators are identified based on knowledge of expected effects between transcriptional regulators and their target genes and, thus, can explain the observed expression changes in our gene dataset. During the IPA analysis, analysis settings included a cutoff for expression *p*-value (set at 0.01), reference set of Ingenuity Knowledge Base (Gene Only) and inclusion of both direct and indirect relationships. The analysis used Fisher's Exact Test to calculate *p*-values.

Alternative splicing events (ASE) were identified by the software *Splicing Express* (Kroll et al., 2015). Briefly, All GTF files created by Cufflinks (Roberts et al., 2011) were inserted with the mouse genome reference sequence (mm10) to run the software *Splicing Express* that is able to identify patterns of simple ASE, including exon skipping, alternative 5′ and 3′ splicing borders and intron retention (Roberts et al., 2011). Because of the software limitation, ASE results from only 122 genes that were selected and identified from the 15 most enriched GO biological process terms were statistically analyzed.

### Other statistical analyses

Student t test was used to detect a statistical significance in MWM performance between the high and low MWM performance. Two way repeated measures ANOVA (group × MWM days) was conducted to test MWM latency in each day followed by the *post-hoc* test of Holm-Sidak method. Normalized gene expression data from DESeq were analyzed by DESeq, PANTHER database, and WGCNA packages, as described above. Briefly, Differential expression (DE) data were generated using DESeq, to investigate significant differences between the groups. Corrected levels of significance and *q*-values were calculated to control the false discovery rate for multiple tests based on the Benjamini-Hochberg equations (Benjamini and Hochberg, 1995).

Normalized gene expression data from DESeq were statistically analyzed by WGCNA that computed all correlation and significance. Pearson product-moment correlation coefficient r was calculated between normalized gene expression data and individual rodents' MWM performance. Hierarchical cluster analysis with a Ward method was used to cluster genes and to provide a summary of selected gene sets. Gene lists from the DE analysis and correlation analysis were analyzed with PANTHER database. In addition, PANTHER statistical over- and under-representation test, by which expression levels of genes in each cluster are statistically tested to be correlated with categories of biological process or molecular function, was performed with gene lists and Log_2_FC data. Best subset regression analysis was used with a selected gene set to identify best regression models. Bland-Altman plot (Bland and Altman, 1986) analysis was used to compare predictive values from regression models with true measured values from RNA-sequencing data and, thus, to evaluate an agreement between the regression models and RNA-sequencing data. All analyses were conducted by using statistical software of JMP® Pro (ver. 11.2.1, SAS Institute Inc.), SigmaPlot (ver. 12.3, Systat Software Inc.) and Microsoft Excel 2013. Alternative splicing event data from the *Splicing Express* (Kroll et al., 2015) were analyzed by Chi-squared and Fisher's exact tests in R (version 3.2.4) to test whether differences of the occurrence in ASE types between the high and low MWM performance groups were significant. Data are represented as mean ± standard error of mean (SEM), and an overall outline for data analyses is shown in Supplementary Figure S1.

## Results

### Behavioral testing and grouping

Twenty-eight mice completed MWM testing over 5 days. The average time to locate the platform across the 5 days was determined (Figure 2A; MWM performance: mean = 24.67, median = 23.82, *SD* = 7.62, range: 26.55, *CV* = 30.90), and utilized to group animals based upon the distribution of their performance. As shown in Figure 2B, there was a significant difference between the means of the high performing mice (*n* = 9) and the low performing mice (*n* = 9). MWM latency in each day also resulted in significant group difference (Figure 2C), but no interaction between group and MWM days was detected. There was a significant group difference in the average of total distance moved during the 5 MWM days (Figure 2D), but no significant difference in the distance moved during the probe trial was detected (Figure 2E). The MWM latency was significantly correlated with total distance moved during the 5 MWM days (*r*^2^ = 0.70, *p* < 0.0001), but no correlations of total distance during probe trial with MWM latency and total distance during the 5 MWM days were detected by Pearson product moment correlation analysis. No statistical group differences were observed on body weight at the time point of tissue collection (5 weeks), average body weight (1–5 weeks) and body weight change from the 1st week to the last week of the experiment (Supplementary Table S1).

### RNA-sequencing analyses

RNA from hippocampal samples from all mice were sequenced. Quantified gene expression data (37,991 genes) from HTSeq analysis were normalized by DESeq, and, then, filtered based on coverage. The distribution of fold changes with *p*-values for these normalized-and-filtered genes (16, 565 genes) is depicted in the volcano plot (Figure 2F). Among them, 521 genes (red-colored dots) resulted in an absolute *r* (|*r*|) value greater or equal to 0.5 computed between gene expression and MWM performance. Most of these genes with |*r*| ≥ 0.5 were implicated in significant group differences in MWM performance based on a typical threshold of significance for RNA-sequencing data (Love et al., 2014).

**Figure 3:**
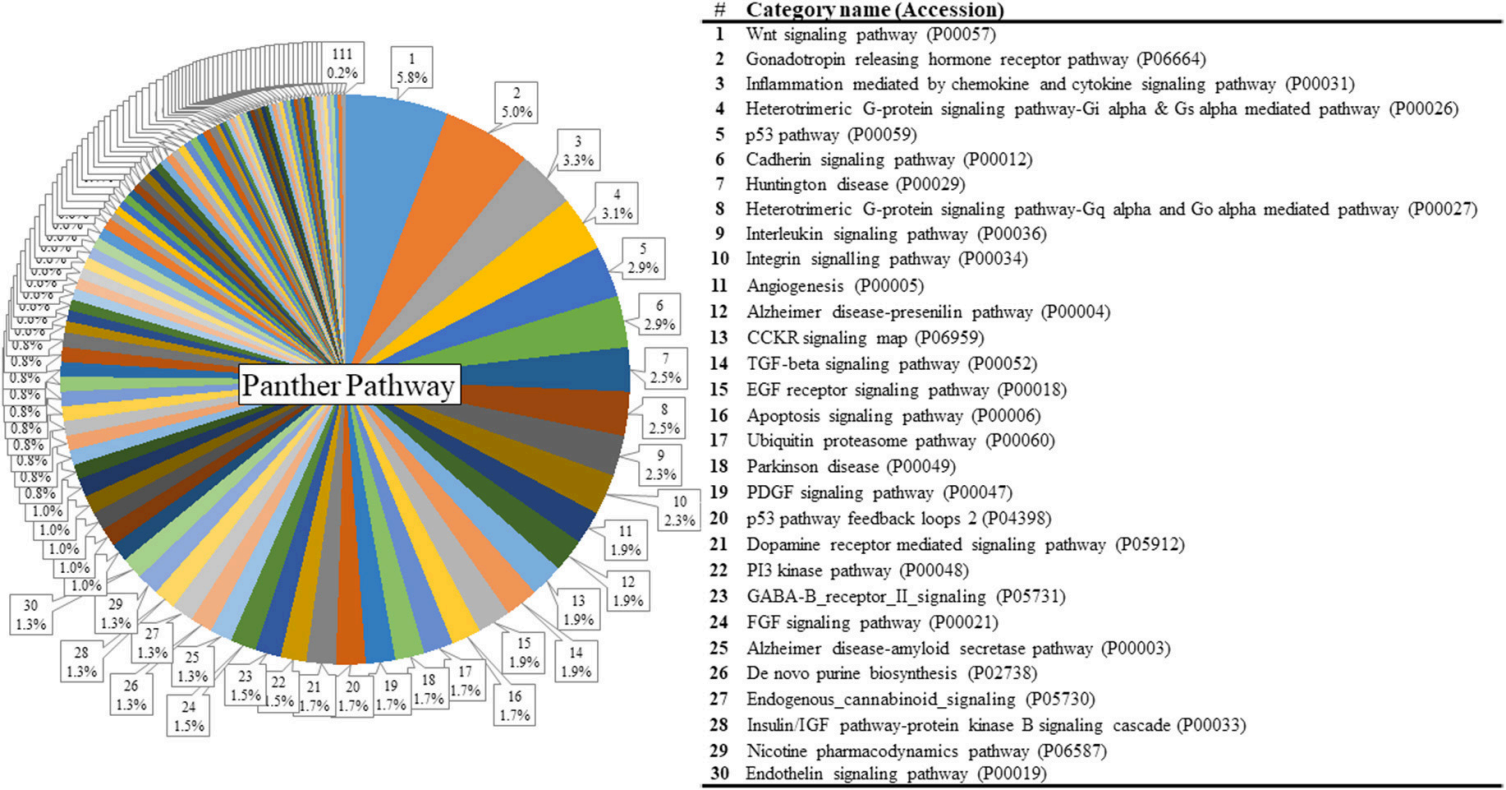
Results of PANTHER pathway database analysis. PANTHER pathway analysis was run with 1250 genes that showed significant group differences and identified 111 pathways figure. The top 30 pathways are listed on the right. For the full index lists of these Panther pathway categories, see Supplementary Table S2.

The list of significant DE genes (*n* = 1,250) were entered into the website of PANTHER pathways analysis, and 111 pathways were identified (Figure 3 and Supplementary Table S2). The PANTHER statistical over- and under-representation test was also performed. The low MWM performance group showed under-expression in some intracellular and neurotransmitter receptor signaling pathways, including metabotropic glutamate, nicotinic acetylcholine, dopamine, corticotropin releasing factor receptor, GABA-B receptor II signaling pathways. The pathways overexpressed by the low MWM performance group included inflammation mediated by chemokine and cytokine signaling pathway, the gonadotropin releasing hormone receptor pathway, wnt signaling pathway, integrin signaling pathway, PDGF signaling pathway, and Alzheimer's disease. The most enriched GO biological processes (Tables 1, 2) that were detected by the PANTHER statistical over- and under-representation test include different receptor signaling pathways, apoptotic-related signaling pathways, immune related pathways and metabolism related pathways. Furthermore, the low MWM performance group showed statistically significant over expression in negative regulation of cell projection organization, negative regulation of neuron projection development, and innate immune response. The statistically significant under represented GO biological processes by the low MWM performance group included, but were not limited to, regulation of molecular function, regulation of apoptotic process, and negative regulation of nucleic acid-templated transcription.

**Table 1:** Functional annotation of genes and 30 most enriched GO biological process terms.

**#**	**GO biological process complete (GO ID)**	**# of genes**	**Over/Under**	***p*-value**
1	Negative regulation of cell projection organization (GO:0031345)	13	+	0.00325
2	Negative regulation of neuron projection development (GO:0010977)	13	+	0.00325
3	Innate immune response (GO:0045087)	35	+	0.00364
4	Cellular response to light stimulus (GO:0071482)	8	−	0.00428
5	Cellular response to UV (GO:0034644)	6	−	0.00433
6	Activation of cysteine-type endopeptidase activity (GO:0097202)	11	−	0.00444
7	Activation of cysteine-type endopeptidase activity involved in apoptotic process (GO:0006919)	11	−	0.00444
8	Carbohydrate homeostasis (GO:0033500)	10	−	0.00616
9	Glucose homeostasis (GO:0042593)	10	−	0.00616
10	Zymogen activation (GO:0031638)	12	−	0.00713
11	Negative regulation of developmental growth (GO:0048640)	8	+	0.00815
12	Regulation of type I interferon production (GO:0032479)	4	+	0.00844
13	Negative regulation of cytokine secretion (GO:0050710)	5	−	0.01160
14	Regulation of cell cycle arrest (GO:0071156)	7	−	0.01180
15	Positive regulation of programmed cell death (GO:0043068)	55	−	0.01190
16	Positive regulation of apoptotic process (GO:0043065)	55	−	0.01190
17	Receptor-mediated endocytosis (GO:0006898)	14	+	0.01240
18	Negative regulation of hemopoiesis (GO:1903707)	11	−	0.01240
19	Regulation of metal ion transport (GO:0010959)	40	−	0.01420
20	Negative regulation of nucleic acid-templated transcription (GO:1903507)	115	−	0.01470
21	Regulation of potassium ion transport (GO:0043266)	10	−	0.01470
22	Cellular response to radiation (GO:0071478)	12	−	0.01620
23	Regulation of protein complex disassembly (GO:0043244)	22	−	0.01720
24	Regulation of collagen biosynthetic process (GO:0032965)	3	−	0.01750
25	Regulation of collagen metabolic process (GO:0010712)	3	−	0.01750
26	Neurotrophin TRK receptor signaling pathway (GO:0048011)	2	−	0.01800
27	Neurotrophin signaling pathway (GO:0038179)	2	−	0.01800
28	Intrinsic apoptotic signaling pathway by p53 class mediator (GO:0072332)	5	−	0.01810
29	Positive regulation of peptidyl-tyrosine phosphorylation (GO:0050731)	13	−	0.01830
30	Regulation of ketone biosynthetic process (GO:0010566)	4	+	0.01830

+/− for Over/Under represent as being toward lower/higher MWM1-5 days performance, respectively

*Genes in the 15 most enriched GO biological process terms are listed in Supplementary Table S6*.

**Table 2:** Functional annotation of genes and 30 greatest number of genes contained in the enriched GO biological process terms.

**#**	**GO biological process complete (GO ID)**	**# of genes**	**Over/Under**	***p*-value**
1	Regulation of molecular function (GO:0065009)	216	−	0.02960
2	Regulation of apoptotic process (GO:0042981)	154	−	0.04270
3	Negative regulation of macromolecule biosynthetic process (GO:0010558)	133	−	0.02740
4	Negative regulation of nucleobase-containing compound metabolic process (GO:0045934)	131	−	0.03610
5	Negative regulation of cellular macromolecule biosynthetic process (GO:2000113)	128	−	0.03880
6	Positive regulation of signal transduction (GO:0009967)	127	−	0.03230
7	Negative regulation of RNA metabolic process (GO:0051253)	120	−	0.02280
8	Negative regulation of RNA biosynthetic process (GO:1902679)	117	−	0.02190
9	Negative regulation of nucleic acid-templated transcription (GO:1903507)	115	−	0.01470
10	Negative regulation of transcription, DNA-templated (GO:0045892)	113	−	0.02660
11	Negative regulation of transcription from RNA polymerase II promoter (GO:0000122)	65	−	0.02260
12	Positive regulation of cell death (GO:0010942)	60	−	0.02280
13	Regulation of catabolic process (GO:0009894)	58	−	0.02480
14	Positive regulation of programmed cell death (GO:0043068)	55	−	0.01190
15	Positive regulation of apoptotic process (GO:0043065)	55	−	0.01190
16	Regulation of metal ion transport (GO:0010959)	40	−	0.01420
17	Innate immune response (GO:0045087)	35	+	0.00364
18	Circulatory system process (GO:0003013)	35	−	0.02790
19	Blood circulation (GO:0008015)	34	−	0.04510
20	Positive regulation of catabolic process (GO:0009896)	32	−	0.04320
21	Aromatic compound catabolic process (GO:0019439)	30	−	0.04880
22	Cellular response to abiotic stimulus (GO:0071214)	25	−	0.02260
23	Regulation of protein complex disassembly (GO:0043244)	22	−	0.01720
24	Cell fate commitment (GO:0045165)	21	−	0.03700
25	Regulation of microtubule polymerization or depolymerization (GO:0031110)	21	−	0.04500
26	Potassium ion transport (GO:0006813)	20	−	0.01960
27	Regulation of protein depolymerization (GO:1901879)	20	−	0.02650
28	Negative regulation of neuron differentiation (GO:0045665)	18	+	0.04760
29	Regulation of microtubule depolymerization (GO:0031114)	17	−	0.02500
30	Positive regulation of peptidase activity (GO:0010952)	16	−	0.02170

*+/− for Over/Under represent as being toward lower/higher MWM1-5 days performance, respectively*.

### Weighted gene-coexpression correlation network analyses

**Figure 4:**
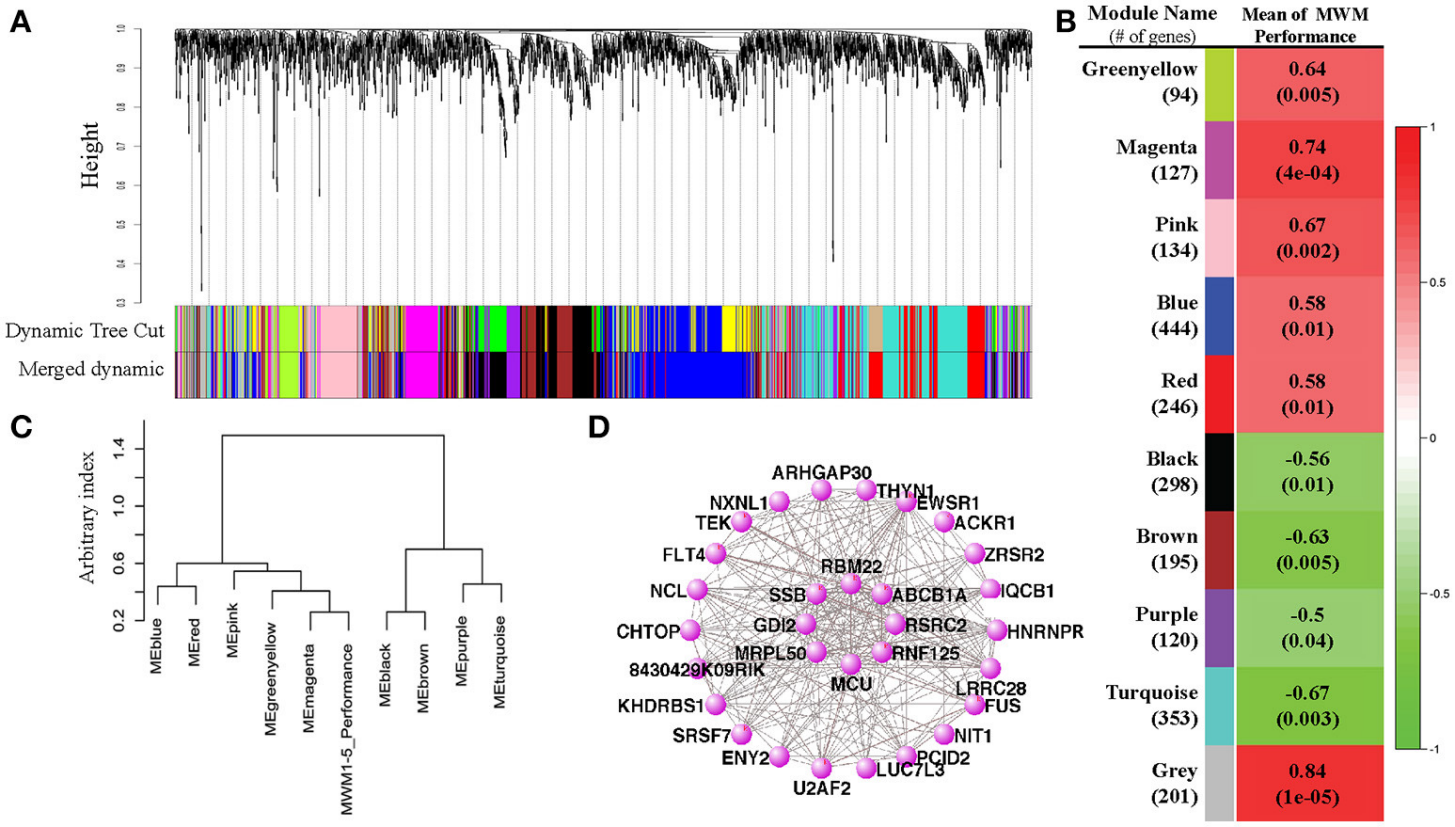
Results of Weight Gene Coexpression Network Analysis (WGCNA). **(A)** shows the clustering dendrogram of genes with dissimilarity based on topological overlap computed by the WGCNA. The original modules and merged modules were colored below the clustering dendrogram. A heatmap created by the WGCNA shows the correlation between the trait MWM performance and each module **(B)**. Pearson *r*-values are depicted on each heatmap block with *p*-values in parentheses. The eigengene dendrogram of the meta-module mutual correlative analysis **(C)** shows how closely the MWM performance was correlated with the modules. The pannal **(C)** shows that the top 2 closest modules with the MWM performance are the magenta and black modules. However, the same meta-module with the trait of MWM performance is the magenta module only. The network connections among the top 30 connected genes in the top highly correlated magenta module with the mean of MWM1-5days performance were visualized by the VisANT software **(D)**. The plots for magenta module show network connections whose topological overlap is above the threshold of 0.02.

To evaluate the differential expression of RNA profiles with MWM performance, we conducted weighted gene-coexpression correlation network analyses (WGCNA). Twelve modules (groups of genes) were merged into 10 based upon the similarity of their expression profiles, known as eigengenes (Figures 4A,B). When the associations between individual genes within each module and the trait (MWM performance) used in this study were quantified, a statistical significance was detected between the modules and the trait of rodent's MWM performance (Figure 4B). All 10 modules resulted in significant correlations with the individual MWM performance: we found positive and inverse correlations with 6 and 4 modules, respectively. The 3 highest correlations were detected by the module-trait association analysis for the magenta (*r* = 0.74, *p* = 0.0004), pink (*r* = 0.67, *p* = 0.002) and turquoise (*r* = −0.67, *p* = 0.003) modules. The meta-modules association analysis, one of WGCNA functions, shows the strength of mutual correlations between modules and the trait, identifying which module has the strongest mutual correlation with the trait. The magenta module showed the strongest mutual correlation with individual MWM performance (Figure 4C). The top 30 connected genes in the magenta module were depicted (Figure 4D), and the plot shows network connections whose topological overlap is above the thresholds of 0.02.

GO enrichment analysis was performed and the 20 best GO terms for each of 10 modules were identified (all listed on Supplementary Table S3). Briefly, among the significant GO terms, the biological process “innate immune response” was detected for both greenyellow and magenta modules. Four different receptor signaling pathways were detected from 3 different modules. GO terms relative to “splice” were detected for the brown and gray modules. The most significant GO terms include granulocyte differentiation (GO:0030851, enrichment *p* < 0.001, # of gene in magenta module = 3), protein targeting to peroxisome (GO:0006625, enrichment *p* < 0.001, # of gene in pink module = 3), mitochondrial transport (GO:0006839, enrichment *p* < 0.001, # of gene in turquoise module = 8), regulation of nitrogen compound metabolic process (GO:0051171, enrichment *p* < 0.001, # of gene in brown module = 32), neuron part (GO:0097458, enrichment *p* = 0.0018, # of gene in turquoise module = 29), and inflammatory response (GO:0006954, enrichment *p* = 0.002, # of gene in turquoise module = 10). The largest number of gene in a module was detected from the blue module: biological process (GO:0008150, enrichment *p* = 0.002, # of gene in turquoise module = 329).

### Analysis of correlation between gene expression and cognitive performance

**Figure 5:**
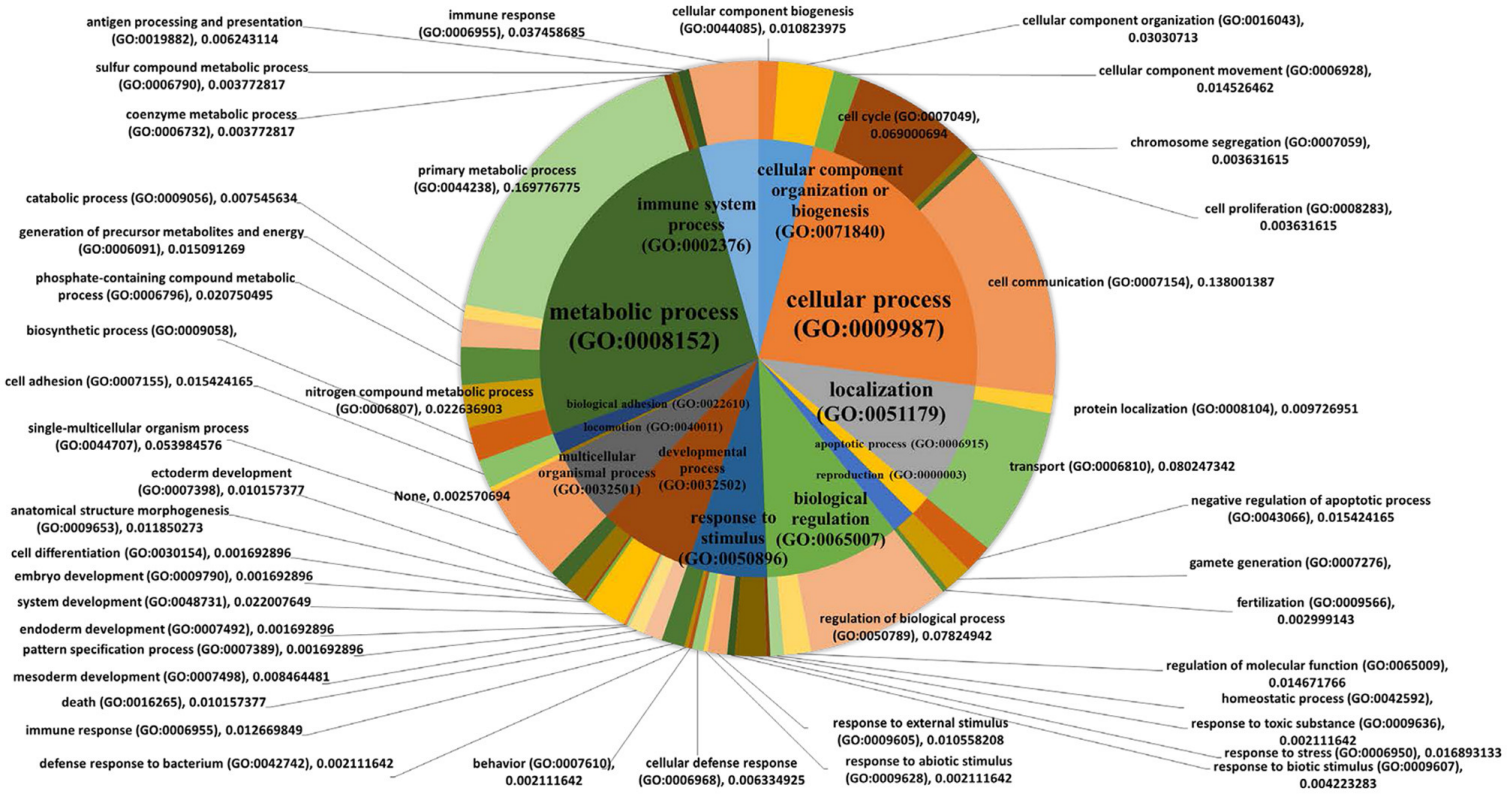
Pie charts showing biological processes. PANTHER biological pathway analysis was used with 411 genes that were significantly correlated with the MWM performance (*p* < 0.050 and |r| ≥ 0.5, Table 1). The 1st order of sub biological processes was drawn as a small pie chart in the center of the main biological process pie chart.

Individuals' MWM performance during 5 days was significantly correlated with the expression of 411 genes (*p* < 0.050 and |r| ≥ 0.5, Table 3). The PANTHER statistical over- and under-representation test was performed, and many metabolic processes were found to be significantly over expressed in the low MWM performance group (Table 4). Interestingly, 5 processes detected are related to RNA regulation: mRNA processing (GO:0006397), RNA metabolic process (GO:0016070), RNA splicing via transesterification reactions (GO:0000375), mRNA splicing via spliceosome (GO:0000398) and RNA splicing (GO:0008380). These 5 RNA regulation-related processes are all significantly overexpressed in the low MWM performance group. Figure 5 shows biological processes with the 1st level of sub-categories, and the top biological processes are similar to the ones from previous analyses: such as, metabolism, cell communication, biological regulation, transport system, and immune system. The hierarchical cluster analysis with the highly correlated genes (*p* ≤ 0.010) resulted in a clear group separation and significant differences in the expression levels of these 82 genes (Supplementary Figure S2).

**Table 3:** Genes that showed significant correlation with MWM_1−5 days_ Performance (*p* < 0.050).

**Absolute correlation coefficient *r***	**Gene symbols**
|*r*| ≥ 0.70	*Dcaf8, Copa, Mkrn3, Ppox, Fcrl6, Slc19a3, Krt9*
0.70 > |*r*| ≥ 0.60	*Ncstn, Gm8113, Ackr1, Exo5, Nr1i3, Nit1, Dennd2c, Gipr, Arr3, Eny2, Ccdc144b, Ticam2, Ndufs2, Lonp1, Fbxl22, Ncl, Ppm1b, Thyn1, Acmsd, Ptger4, Cdk11b, Nostrin, Icosl, 4930513N10Rik, Ccno, Gm14634, Ldhd, 9530051G07Rik, Sdhaf3, Hmgcll1, Exosc10, Fbxo33, Snupn, Gm11620, Cdc25a, Medag, Scd4, Eno1, Ubap1, Tbx15, Fmo2, Asun, Stag3, Kcp, Xlr3a, Ccdc63, Scn9a, Sbsn, Ikzf5, Cttn, Msl3, Zbtb12, Wnt6, AU019823, Vsig10l, Ifi202b, Cthrc1, Atpaf1, Khdrbs1, Kbtbd4*
0.60 > |*r*| ≥ 0.50	*Eif4h, 1700028J19Rik, Fos, Wscd2, Nr1d2, Camkv, Hsd3b3, Polm, Frmd5, Tor1b, Tymp, Pvr, Aim2, Ttk, A230072E10Rik, Gm10226, 2410002F23Rik, Megf6, Cwh43, Slc2a5, Ict1os, Exoc3l2, Diaph3, Capn10, Cd33, Bcl2l15, Fxyd5, Gm15720, Lrrc28, Rbm8a2, 1700067K01Rik, Atp2c2, Ncald, Chtop, Cxcl5, Mcidas, Fcgbp, Cited2, Rbm22, Gm10451, Hps1, Dlst, Sphk1, Mms22l, E2f6, Tmco6, Abhd10, Gm15610, Tma7-ps, St13, Fam188b, Gapdhs, Plau, Tmem50b, Frmd7, U2af2, Gm14735, Ly6c1, Fgfr1op, Sgpp2, Nle1, Slamf8, Smad1, Xbp1, Cbln4, Gpatch1, Dnm3, Fcna, Abcb1a, Slc11a1, Wdsub1, Tmem135, Zfp180, Tmem55a, 9430015G10Rik, Thbd, 1110008L16Rik, Fam126a, Stx3, Mcu, Igsf8, Dusp1, Ccdc184, 2810006K23Rik, Cplx1, Cse1l, Dclre1a, Srsf5, Whamm, Cnih4, Ewsr1, Fbxo3, Trp63, Col22a1, Hnrnpa1, Irf1, Kcnab3, Slc26a1, Wdyhv1, Liph, Sigirr, Mybpc2, Dars2, Zfp772, 1700001K19Rik, Uspl1, Cir1, Tmem62, Frmd3, Dalrd3, Ttc37, Gm22875, Mzt1, Lrrd1, P2rx6, Xrn2, Palb2, Ocel1, Il5ra, Sec22b, Gm10785, Pak4, Luc7l3, Gm12892, Rbm43, Gch1, Raver1, 2210406O10Rik, Siah2, Hdac1, Abcc3, Hn1l, Pfkfb4, Slc25a40, Xlr3b, Slc9a6, Dmrta2, Fbxw11, Susd2, Gm13031, 8430429K09Rik, Alg1, Gpr3, Zbtb37, Arl8a, Rbm10, Gm12158, Ears2, AA414768, Nudt19, Slc38a2, Bnip3l, Rcor2, Ust, Fam103a1, Tma16, Slc2a1, Ankrd34c, Taf15, Smpd4, Cyth1, Zrsr1, Fxyd3, Hspb1, 1500017E21Rik, Ccdc173, Slc35f6, Hspa12b, Nek6, Islr2, Trmt2a, Zkscan6, Fgf17, Esam, Aspdh, Gm14569, Fancd2os, Sft2d2, Mfn2, Tek, Ier3, Gm14257, Dcaf12l2, Ccdc110, Sirt7, Ythdf1, Ldb1, Itpka, Zcchc16, Clpb, I830077J02Rik, Hrk, Eno1b, Mgat4b, Clec10a, Pdp1, Pkd1l3, Vps37c, Zfp94, Tubb3, Six1, Syt4, Rab3d, Tldc1, Chst5, Btg3, 4930538K18Rik, Rnf125, Armcx1, Ctu1, Ppp1cc, Gm12279*
|*r*| < 0.5 (the lowest *r* = 0.47)	*Atl2, H3f3aos, Gm12500, Rbmx, Krt73, Flt4, Gsx1, Pnldc1, Pgpep1, Atg4a, Fus, Miip, Ccnjl, Dmwd, Nudt3, Xdh, C1qtnf9, Gm5187, Polr1a, Matn2, Herpud2, Cdc25c, Dach2, 3930402G23Rik, Gm2710, Lrrtm2, Ripply3, Kcnj5, Vsx2, Gm10564, Trim11, 4921507P07Rik, Snord35b, Olfm3, Tmem167b, Hsf1, Rsrc2, Alyref2, Sars2, Gm15728, Ccnb2-ps, Tbce, C530005A16Rik, Homer3, Gm14246, Gm13344, Snord91a, Lynx1, Xcr1, Cdh12, Defb1, Elp3, Ttc38, Impdh1, Trim71, Pxdn, Gm4735, Lsmem1, Gm1840, Aspn, Xrcc5, Tubgcp5, 4933427I04Rik, Chrna2, 9330158H04Rik, Pip5kl1, Dnd1, Gm12059, Tmem143, Pfn4, Zdhhc2, Fam71e1, Adm, Mrrf, Pik3ca, Odf2, Mael, Trib1, Brinp3, Rhbdl2, Traip, Adgrl4, Lag3, P2rx4, Hspa2, Nlrx1, Zfp961, Tmem33, Pawr, 1700088E04Rik, Lnp, Clcn4, Rab2a, Ago4, Tmem65, Spn, Misp, Thtpa, Sctr, Gsg2, Grhl1, Gm8898, Glrb, Srsf6, Gosr2, B3gnt3, Mapk6, Slc16a11, Slc25a47, Fbxl5, Gm6206, Nvl*

**Table 4:** GO-Slim Biological Process Results of Statistical overrepresentation test by the PANTHER with 410 significantly correlated genes (input: genes with EntrezGene ID, *p* < 0.050, and |*r*| ≥ 0.5).

**PANTHER GO-slim biological process (GO ID)**	**Over/Under**	**Fold enrichment**	***P*-value**
Pteridine-containing compound metabolic process (GO:0042558)	+	71.77	1.38E-02
Glycolysis (GO:0006096)	+	9.90	7.93E-04
Response to abiotic stimulus (GO:0009628)	+	6.24	4.15E-02
Synaptic vesicle exocytosis (GO:0016079)	+	4.10	1.73E-02
Nucleobase-containing compound transport (GO:0015931)	+	3.36	9.72E-03
RNA splicing, via transesterification reactions (GO:0000375)	+	3.24	1.16E-02
RNA splicing (GO:0008380)	+	3.17	1.28E-02
mRNA splicing, via spliceosome (GO:0000398)	+	3.07	5.18E-03
Spermatogenesis (GO:0007283)	+	2.66	2.72E-02
mRNA processing (GO:0006397)	+	2.64	5.38E-03
Phospholipid metabolic process (GO:0006644)	+	2.43	3.92E-02
RNA metabolic process (GO:0016070)	+	1.4	1.43E-02
Nucleobase-containing compound metabolic process (GO:0006139)	+	1.34	8.17E-03
Primary metabolic process (GO:0044238)	+	1.21	7.93E-03
Metabolic process (GO:0008152)	+	1.15	2.09E-02
Unclassified (UNCLASSIFIED)	−	0.83	6.09E-03
Regulation of molecular function (GO:0065009)	−	0.44	8.38E-03
Sensory perception (GO:0007600)	−	0.34	2.36E-02
Regulation of catalytic activity (GO:0050790)	−	0.32	1.37E-03

*+/− for Over/Under represent as being toward low/high MWM performing group, respectively*.

### Ingenuity's upstream regulator analysis

**Figure 6:**
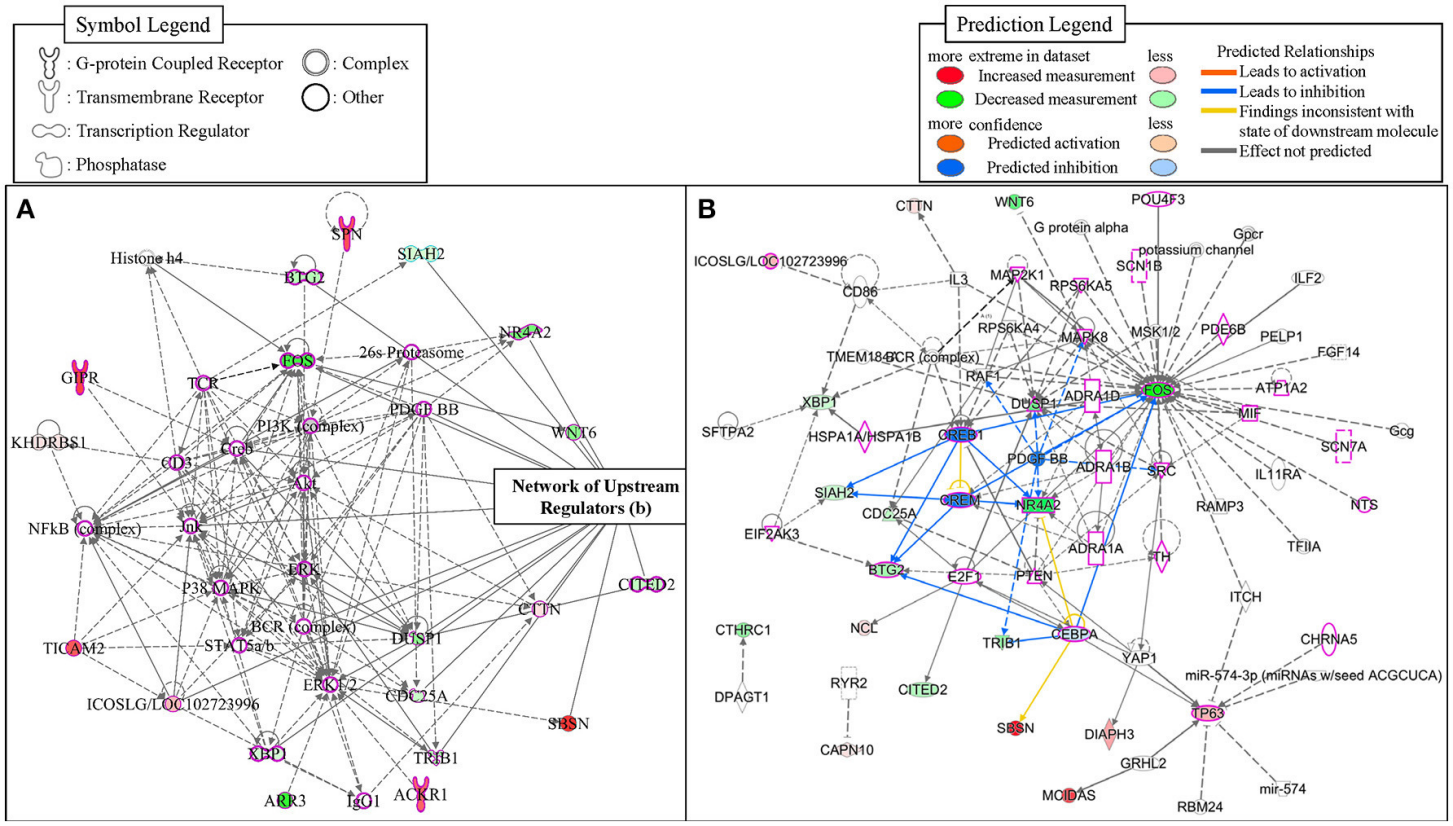
The networks identified from the IPA. The network 1 **(A)** was depicted with 35 molecules. The network of upstream transcriptional regulators identified by the Ingenuity's Upstream Regulator Analysis **(B)**. The regulators that were identified to be associated with learning behaviors were outlined with pink-color.

To identify potential transcriptional regulators that can explain the expression changes in our genes between the low and high MWM performance groups, the Ingenuity's upstream regulator analysis was conducted. The Ingenuity's upstream regulator analysis identified 16 different molecule types and 247 upstream transcriptional regulators (all listed on Supplementary Table S4). Among them, 7 cytokines (IL1B, IL3, IL5, LIF, MIF, TNF, and TNFSF13B) were identified and the reduced activation was observed from IL1B (activation z score = −1.954, p for overlap = 0.041). Nicotinic acetylcholine receptor was also identified and negative activation z score (−1.997) was calculated for this (p for overlap = 0.001). Thirty four transcriptional regulators were identified, but activation z scores for only CREB1 (−1.969) and CREM (−1.982) were identified with significant *p*-values for overlap (0.002 and 0.001, respectively). Network for upstream regulators was created (Figure 6) and 28 regulators were identified to be associated with learning behavior (outlined with pink color). Among these 28 regulators, CREB1 and CREM regulators were predicted as inhibition and TP63 and ICOSLG regulators were predicted as activation. When overlaid with biological functions, 15 upstream regulators were significantly associated with learning behavior (*p* = 2.43E-11), and 23 upstream regulators were significantly associated with nervous system development and function (*p*-value range: 1.32E-7–6.51E-7). When overlaid with diseases, neurological diseases, including Alzheimer disease (*p* = 5.02E-7), were identified to be significantly associated with 38 upstream regulators (*p*-value range: 1.4E-11–5.13E-6). Additionally, the network showed that FOS, NR4A2, WNT6, and DUSP1 regulators were highly decreased in our gene dataset. With our gene dataset, the IPA identified 5 networks (score ≥ 10, Table 5). The network 1 contained 17 upstream transcriptional regulators (Figure 6). Some upstream transcriptional regulators were also identified from the networks 2 (NCL), 3 (TRIB1). 4 (DIAPH3, GRHL2, MCIDAS, TP63), and 5 (CAPN10, CHRNA5, CTHRC1, RYR2).

**Table 5:** Top 5 Networks identified by the Ingenuity® Pathway Analysis (IPA).

**ID**	**Molecules in network**	**Score**	**Focus molecules**
1	26s Proteasome, ACKR1, Akt, ARR3, BCR (complex), BTG2, CD3, CDC25A, CITED2, Creb, CTTN, DUSP1, ERK, ERK1/2, FOS, GIPR, Histone h4, ICOSLG/LOC102723996, IgG1, Jnk, KHDRBS1, NFkB (complex), NR4A2, P38 MAPK, PDGF BB, PI3K (complex), SBSN, SIAH2, SPN, STAT5a/b, TCR, TICAM2, TRIB1, WNT6, XBP1	36	19
2	ACOX1, BCL2, Cd33, CD68, CD84, CDK9, CDKN1C, CEBPD, COL4A1, COPA, cytochrome C, DIO1, E2F3, GH1, Growth hormone, IGF1R, IGFBP5, IRS2, LIPE, LONP1, LPL, Mcpt8, MEDAG, MYBL1, MYBL2, NCL, NCSTN, NDUFS2, NIT1, PPM1B, PRKAR1A, PTTG1, RB1, STAG3, TXN	16	10
3	ADAMTS1, AGMAT, Cg, CNIH4, CYP17A1, DUSP10, EBI3, F2RL1, FSH, GAD1, GLI1, Histone h3, HMGA2, HPGD, IER3, IGFBP7, IL4, IL11, IMPDH1, KDM5B, KMT2D, LGALS3BP, LMNB1, POLM, PPOX, PTX3, RETN, RPS6KA3, SMARCA4, SNUPN, STAR, TBX15, Tpm4, TRIB1, WDSUB1	14	9
4	BCAR3, CDK11A, DIAPH3, ELK3, ENO1, ERK, ETS1, ETV4, GRHL2, HAS3, INF2, ITGA3, KIF23, LAMA3, MCIDAS, MKRN3, MYH6, MYL9, PERP, PHLDA1, PIK3R3, S100A7, S100A8, SCN9A, SEMA3B, SLC26A1, SLPI, SRF, TLR3, TNFAIP8, TP63, Tpm1, ULBP2, YY1, ZDHHC2	14	9
5	ARAF, ASPM, CAMK4, CAPN10, CBLN4, CCR7, CDK2AP1, CHRNA5, CPLX1, CTHRC1, DDC, DOCK1, EP300, FKBP1B, FOXO1, HNF4A, IKZF5, INSIG1, INTS13, IRS2, KIF1B, LIPE, LYL1, MED1, NKX3-1, NR1I3, NTN1, Proinsulin, RAB3A, RYR2, SERPINB2, SNCA, SREBF1, TAL1, TCF3	10	7

### Alternative splicing events

**Figure 7:**
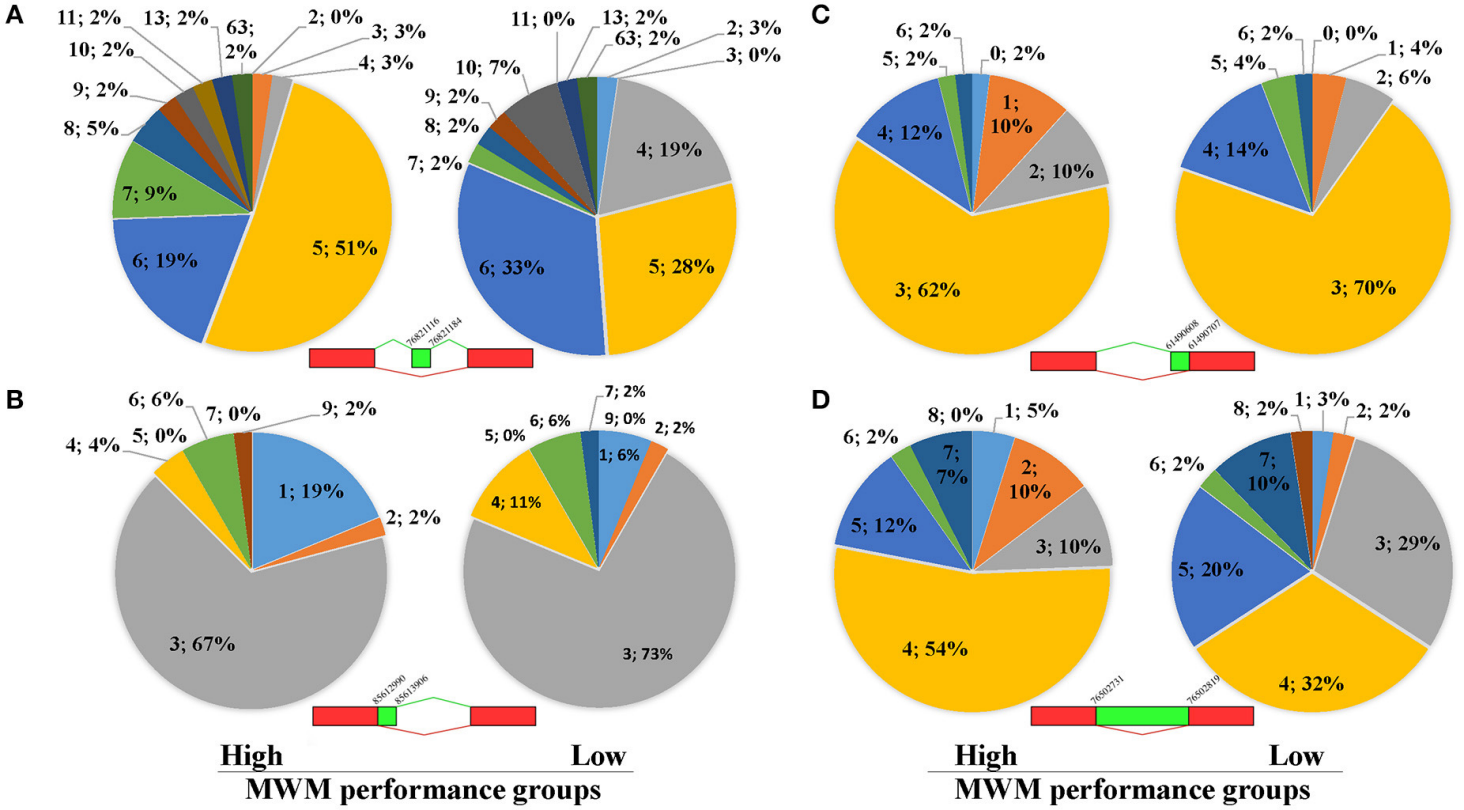
Results of alternative splice events for 122 genes listed on Supplementary Table-S1. The pie charts **(A)** represent exon skipping events for genes with more or equal to 5 events of exon skipping. There is a significant difference in the Exon Skipping Events between the groups (*p* = 0.043) detected by Fisher's Exact Test. **(B)** represents data for alternative 3' splice border for genes with more or equal to 3 events of alternative 3' splice borders. There is no significant difference between the groups (*p* = 0.82) detected by Fisher's Exact Test. **(C)** shows data for alternative 5' splice border for genes with more or equal to 3 events of alternative 5' splice borders. There is no significant difference between the groups (*p* = 0.32) detected by Fisher's Exact Test. **(D)** shows data for intron retention for genes with more or equal to 4 events of intron retention. There is no significant difference between the groups (*p* = 0.12) detected by Fisher's Exact Test. Each data (N; %) on the figures represent as the number of alternative splicing events and percentiles, respectively.

Because unique biological processes identified a potential contribution of RNA splicing to group differences (Table 4), analyses of ASE were carried out using the recently developed software *Splicing Express* (Kroll et al., 2015). No statistical differences were detected in the number of Genes (X-squared = 0.81714, *p* = 0.85) and the number of Total Events (X-squared = 1.4336, *p* = 0.70) between the high and low MWM performance groups (Supplementary Table S5). Because of the difficulty in pulling out all ASE data from the Splicing Express, 122 genes in the 15 most enriched GO biological process terms (Supplementary Table S6) were chosen to analyze ASE. Overall, similar patterns in the ASE types between the groups were discovered (Supplementary Figure S3). However, we found group differences in the ASE exon skipping type in genes (Figure 7A, *p*-value from Fisher's exact test was 0.043). The low MWM performance group has more genes with 4, 6 and 10 exon skipping events but fewer genes with 5, 7, and 8 exon skipping events. Alternative 3′ and 5′splice sites and intron retention for these genes are depicted on Figures 7B–D (*p*-values from Fisher's exact test were 0.82, 0.32, and 0.12, respectively). Because the Figure 7D showed a visually greater difference in certain intron retention events (especially 2, 3, 4, and 5 events), the Fisher's exact test was run with only 1 through 5 intron retention events, then a statistical significance was detected (*p* = 0.048). These ASE data analyses suggest that the number and types of ASE for some genes were significantly different between the low and high MWM performance groups.

### Predictive regression models and evaluation of the regression models

**Figure 8:**
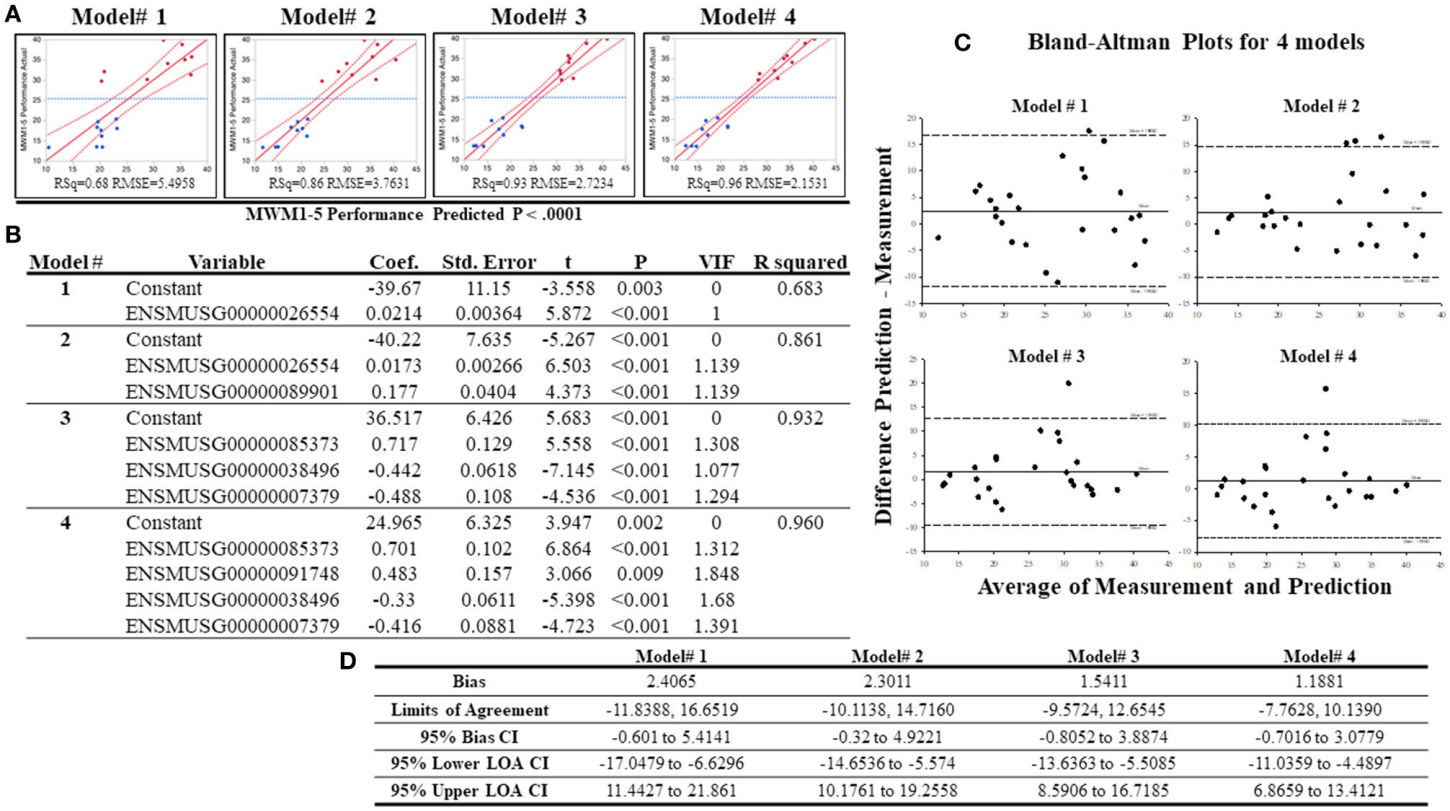
Results of the best subset regression analysis. Four acceptable regression models **(A)** for mouse MWM1-5days performance were identified and are shown here with a regression line (solid red line) and confidence of fit curves (curved red dot lines). Blue dashed lines are the mean of the MWM performance for the 5 days (MWM1-5 performance). Statistical information for the 4 regression models are shown on **(B)**. Bland-Altman Plots with 18 samples in the low and high groups in addition to 7 additional samples (total *n* = 25). **(C)** highlights the center lines represent bias of each model and upper and lower dot lines are 95% upper and lower limits of agreements. **(D)** shows statistical information of Bland-Altman Plots for acceptable 4 regression models created by the best subset regression analysis.

To analyze whether individual's MWW performance could be predicted from the expression of selected genes, best subset regression was performed with the top highly correlated genes. This allows the identification of the best regression model for cognitive performance under chronic stress. From the top 16 genes, the best subset regression method generated 10 potential regression models (from a total of 16 models, Supplementary Table S7). Variance inflation factors (VIFs) of each model were computed and excluded the models 5–10 due to VIFs > 2 although these models presented higher *r*^2^-values than the remaining 4 models. The 4 regression models (0.68 ≤ r^2^ ≤ 0.96) chosen are depicted with their statistical information in Figures 8A,B. To validate these models, we added additional whole transcriptome RNA-sequencing data from 7 different strains (average performing strains) that were excluded from grouping, and drew a Bland-Altman (BA) Plot to analyze the agreement between predicted values (from the equations obtained from the 4 regression models) and measured values (from normalized DESeq data). As shown on Figures 8C,D, bias ranged from 1.18 to 2.41, and at least one sample from all models was detected above the 95% upper limits of agreement CI. Among these models, the model# 4 showed the smallest bias (bias = 1.19, 95% Bias CI: −0.70 to 3.08) with the smallest range of limits of agreement (−7.76, 10.14), and the differences of 24 samples (out of 25 samples; thus, 96%) between the prediction and measurement values were positioned between the 95% upper and lower limits of agreements.

## Discussion

Our main research question for this study was whether a divergence in MWM-dependent cognitive performance under chronic stress was associated with transcriptomic changes in the mouse hippocampus. If so, then our next questions were which genes were related and which biological signaling pathways may be explainable for the variance in cognitive function under chronic stress. To answer our research questions, we conducted whole transcriptome RNA-sequencing analyses for hippocampal samples from BXD recombinant inbred mice that had completed behavioral testing under chronic stress conditions. Our data show that a variety of MWM-dependent cognitive performance of mice that received the same amount of stressors is associated with the expression profiles of not all, but some genes in hippocampus. Analysis of RNA-sequencing data resulted in the identification of biological pathways that were related to the level of behavioral performance. In sum, our results show that hippocampal dependent spatial memory performance under chronic stress is related to dynamic differences in hippocampal gene profiles and their corresponding biological pathways.

In this study, we utilized multiple methods to analyze and interpret whole transcriptome RNA-sequencing data, including differential expression (DE) analysis, WGCNA, Ingenuity's upstream regulator analysis and expression comparison of the genes that were highly correlated with MWM-dependent spatial memory in addition to the use of the web-based database (PANTHER) to identify biological pathways. Our RNA-sequencing data from the DE analysis between high and low MWM performance showed that 1,250 genes were differentially expressed (Figure 2F). To understand the biological function of these genes, PANTHER pathway analysis and statistical over- and under- representation test were used and identified 111 pathways.

The Wnt signaling pathway (P00057) was recognized as the top pathway by PANTHER pathway analysis (Figure 3). This pathway is known to be associated with hippocampal neurogenesis and dysregulation of the wnt signaling pathway in hippocampus was previously reported to impair hippocampal dependent cognitive performance in rats (Jessberger et al., 2009). The PANTHER over-/under- representation test showed that the wnt signaling pathway is overexpressed in the low MWM performance group, but no statistical significance was detected. Although literature showed the inhibition of WNT signaling in the dentate gyrus prevented the neurogenesis in hippocampus, dysregulation of this signaling was also reported to impair cognitive performance (Lie et al., 2005; Jessberger et al., 2009). Therefore, although our data showed over-expression in the Wnt signaling pathway in the low MWM performing group, there is a possibility that over-expression of the Wnt signaling in the low MWM performing group may be resulted of dysregulation of the pathway. Another possibility for connecting Wnt signaling pathways to cognitive performance may be explainable by a role of Wnt signaling in cellular metabolism (Komiya and Habas, 2008; Abiola et al., 2009). One of Wnt signaling downstream effects is to indirectly regulate mammalian target of rapamycin (mTOR) signaling pathway, and the expression of mTOR signaling also regulates with cellular homeostasis with regulation of energy, oxygen and growth factor (Komiya and Habas, 2008; Sengupta et al., 2010). The PANTHER pathway analysis (Supplementary Table S2) in this study also identified some metabolism pathways, such as Insulin-related pathways (P00033 and P00032), Glycolysis (P00024) and Pentose phosphate pathway (P02762). Especially, our data (Table 4) showed that metabolism-related signaling pathways were significantly modified by chronic stress. Therefore, it may be possible that chronic stress resilience/vulnerability may be associated with the Wnt signaling pathways through the changes in mTOR signaling and metabolisms in the hippocampus, but future study on this possibility should be guaranteed.

To better understand our data on the WNT signaling, we carefully investigated the expression of WNT signaling-associated genes from the transcriptomic data. We found that the gene Wnt6 showed a significantly decreased expression (log_2_FC = −0.54, *p* < 0.01) in the low MWM performance group. Because the PANTHER over-/under-representation test use the expression of all relative genes, the overall overexpression in the wnt signaling pathways was resulted although the expression level of Wnt6 gene was significantly decreased. Therefore, careful investigations on WNT signaling-associated molecules should be granted. The next top pathway detected was the gonadotropin releasing hormone receptor pathway (P06664), which is known to regulate hippocampal spine density (Prange-Kiel et al., 2008). Dysregulation of hippocampal expression of gonadotropin releasing hormone receptor is also associated with depressive mood symptoms (Walf and Frye, 2006). The PANTHER statistical test showed an increased expression, although it did not reach statistical significance, of these 2 pathways in the low MWM performance group. As seen on the Wnt6 gene, the investigation of individual genes in these signaling pathways resulted in the identification of specific genes that were differentially expressed between the high and low performing groups.

The inflammation mediated by cytokine and chemokine signaling pathway (P00031) was also detected as one of top pathways. Accumulating evidence reports that inflammation is boosted by chronic stress and plays an important role in stress-induced behaviors and cognitive performance (Hodes et al., 2015; Jung et al., 2015; Rickenbach et al., 2015; Cuadrado-Tejedor and Garcia-Osta, 2016). Our data show an increase of cytokine and chemokine-mediated inflammation pathways, identified with 23 genes, in the low performing group. These data provide additional support that hippocampal-dependent cognitive outcomes under chronic stress environments are associated with dynamic differences in hippocampal gene profiles and biological pathways.

To further understand the relationship between MWM-dependent cognitive performance and hippocampal whole transcriptome RNA-sequencing data, we performed WGCNA. WGCNA generated 10 different gene groups, referred to as modules (Figure 4). We first investigated correlations with strain means of MWM performance using these modules. All 10 modules, including the gray module that contains any gene that does not show a correlation with any gene groups, resulted in a significant correlation with the MWM performance. The results show 6 positive and 4 inverse correlations between MWM performance and modules, suggesting that the expression of gene profiles in each module is significantly associated with the MWM performance. The highest correlated module (magenta) was further analyzed, and network connections of the top 30 genes from the magenta module were investigated. Of the top 30 genes in the magenta module, the top PANTHER GO-Slim biological process was the metabolic process followed by single-multicellular organismal process, transport, cell process, biological regulation, immune system. These data suggest that cognitive performance under chronic stress is highly associated with modifications in metabolic, cellular organismal and transport pathways in the hippocampus. Some of these processes, such as metabolic, cellular, biological, and immune processes, are also identified from other modules.

The analysis of PANTHER GO-Slim biological process was additionally performed with all genes from the magenta module, thereby identifying 12 different biological terms, with the top 5 terms being metabolic process (GO:0008152, 41 genes), cellular process (GO:0009987, 32 genes), biological regulation (GO:0065007, 20 genes), biological categories of response to stimulus (GO:0050896, 12 genes) and immune system process (GO:0002376, 7 genes). These findings from the top 30 and all genes of the highest correlated module are also supported by the GO enrichment analysis with genes from other modules. The GO enrichment analysis of the WGCNA package, performed with genes in the modules, identified the 20 best GO terms for each module (Supplementary Table S3). The majority of these GO terms from the WGCNA analysis are associated with metabolism, cellular process, neurotransmitter, and receptor regulation, and immune response. These results from the WGCNA analysis suggest that chronic stress environments modify different pathways in hippocampus that may depend on hippocampal dependent cognitive performance.

We narrowed down the WGCNA results by performing the PANTHER statistical overrepresentation test with the genes whose expression were significantly and highly correlated with MWM performance. The hierarchical cluster analysis identified 82 genes (Supplementary Figure S2). In the low MWM performance group, 46 genes were significantly over-expressed while 36 genes were significantly down-regulated when compared to the high performing group. Once again, most genes were related to metabolism, cellular process, and transport system. Moreover, many of these genes were previously reported to be associated with cognitive impairment and diseases/disorders, including Alzheimer's, Parkinson's, epilepsy, cortical myoclonus, and cancers (see the literature list on the Supplementary Table S8). Additionally, our data from the IPA analysis also provided evidence supporting that many upstream regulators, identified from our gene dataset, were significantly associated with cognitive performance and neurological diseases. These previously published evidence, in conjunction with our data reported here, suggest that the chronic stress-induced reduction in cognitive performance may be associated with a higher incidence of neurological diseases/disorders (literatures listed on the Supplementary Table S8). This possibility may be supported by previous findings, showing that stress is one of the main contributors to cognitive impairment (Johnson, 2015; Rickenbach et al., 2015) and Alzheimer's disease (Cuadrado-Tejedor et al., 2012; Cuadrado-Tejedor and Garcia-Osta, 2016) in addition to other neurological diseases and disorders (Heller et al., 2014; Gelisse et al., 2015; Hodes et al., 2015; Mcewen et al., 2015). However, further investigation into effects of individual genes on cognitive impairment and other diseases and disorders under chronic stress are warranted in order to confirm our findings.

We investigated ASE between the high and low MWM performance groups. When 122 genes reported from the 15 most enriched GO biological process terms were analyzed, we found that there were significant group differences in the most common types of ASE, such as exon skipping. Exon skipping events occur in all cells to regulate biological function through the generation of protein isoforms with distinct biological function, such as enhancing, reducing, differing and opposing enzymatic activities (Cooper et al., 2009; Ye et al., 2014). The variability of ASE is known to be associated with diseases/disorders and has a significant impact on individual variability of disease severity and drug responses (Wang and Cooper, 2007; Cooper et al., 2009). Recent publications also demonstrated that alternative splicing is associated to behavioral outcomes. For instance, Martin et al. (2013) reported that an alternation of RNA splicing is associated with behavioral changes in a mouse model and highlighted the role of RNA splicing in rodent behaviors (Martin et al., 2013) and, possibly, cognitive performance. This finding was also supported by another group (Rittschof et al., 2014). Therefore, our findings together with previous evidence suggest that the wide variance of ASE is associated with the cognitive ability to adapt to chronic stress and a divergence in memory performance under stress. However, future studies are warranted to further investigate this possibility.

Finally, we addressed whether RNA-sequencing data could predict MWM performance using regression models. The best subset regression analysis was used with the 16 highest correlated genes, and, among 16 different models, it generated 4 potentially reliable models with the r^2^ range of 0.68–0.96 to estimate and predict the MWM performance under chronic stress. To validate these regression models, additional RNA-sequencing data were used from all animals, thus, including the low, high and middle performing groups all. The predictive data from the regression models were compared to measured expression values from 25 RNA-sequenced samples (*n* = 9 high performing, *n* = 9 low performing, *n* = 7 middle performing). This BA plot suggests that a regression equation of the model# 4 (*r*^2^ = 0.96) may be useful to predict cognitive performance under prolonged stressful environments. Although the regression model showed a significantly strong correlation, it would be necessary to retest our regression models with larger sizes of sequencing or gene samples and to identify other gene sets that are potentially used to predict the cognitive performance under chronic stress. Furthermore, our results highlight multiple potential targets for future research to better elucidate roles of specific genes/proteins during cognitive function in a chronic stress environment.

In this study, because of the innate nature of whole RNA transcriptomic data, a great number of biological signaling pathways were identified from our transcriptomic data, such as wnt signaling, gonadotropin releasing hormone receptor pathway, inflammation signaling, metabolic and immune pathways. These results showed that individual difference in MWM-based cognitive function under chronic stress were significantly associated with modifications of these signaling pathways. The modifications of these signaling pathways detected from our transcriptomic data may be caused by the dysregulation of stress pathways, especially hypothalamus-pituitary-adrenal (HPA) axis, induced by the exposure to chronic stress environments. Stressor activates the HPA axis and stimulates releasing a number of factors, including hormones catecholamines and corticoids. These stress-induced hormones evoke the fight-or-flight response, promote short-term adaptation, known as allostasis, and may produce adaptive effects on these systems (Mcewen and Gianaros, 2010). However, if stress pathways are activated by chronically stressful events, known as allostatic load, the released levels of stress-induced hormones will be dysregulated and biological sensitivities to these hormones become blunted. Consequently, dysregulation of these hormones, induced by allostatic load, produces maladaptive effects on biological systems, especially metabolism, immune, inflammation, cardiovascular, endocrine and central nervous systems (Selye, 1976; Besedovsky and Del Rey, 1996; Mcewen and Gianaros, 2010; Iwata et al., 2013; Mcewen et al., 2015) that were identified from our transcriptomic data and support our findings in this study. However, additional analysis of the relationship between stress response (or coping strategy) and cognitive performance could extend the observations of this study. Therefore, future study is needed to elucidate underlying mechanism, by which these signaling pathways are modified by chronic stress. If possible, it is necessary to identify which molecules in each pathway play a critical role in cognitive function under chronic stress. Moreover, because we used only one cognitive test (MWM test) to evaluate cognitive function in this study, it is strongly recommended for future investigators to use more behavioral test methods to assess cognitive function.

## Conclusions

Human beings are constantly exposed to multiple sources of stress that negatively affect cognitive performance and the brain, especially hippocampal regions (Chrousos and Gold, 1992; Schmidt et al., 2008; Chrousos, 2009; Babenko et al., 2015). Our results examining the relationship between stress susceptibility, hippocampal gene expression and spatial memory performance aid our understanding of individual performance variability in a high stress environment. These results show that unique gene expression profiles and pathways within the hippocampus are related to spatial memory performance following a chronic stress environment. The specific targets identified in this study should provide the basis for future studies examining the relation between stress susceptibility and cognitive function. In summary, the divergence in spatial memory performance, measured by the MWM test, in a high stress environment is related to the biological pathways modulated in the hippocampus.

## Data access

The datasets generated and/or analyzed during the current study are available from the corresponding author on reasonable request.

## Author contributions

SJ designed the research, generated the RNA-sequencing data set, conducted the bioinformatic analyses, interpreted all data and wrote manuscript. MB was involved with data interpretation and manuscript writing. MP completed the RNA sequencing and contributed to analysis and manuscript writing. RJ designed the research and supervised all data production and manuscript preparation.

### Conflict of interest statement

The authors declare that the research was conducted in the absence of any commercial or financial relationships that could be construed as a potential conflict of interest.

## Supplementary material

Supplementary Figure S1 The brief summary of data analysis methods for all tables and figures reported in the manuscript.

Supplementary Figure S2 Hierarchical Cluster with Dendrogram (Method = Ward) for significantly correlated genes with MWM_1−5*days*_ Performance (# of genes = 82, *p* ≤ 0.010).

Supplementary Figure S3 Results of alternative splicing event types in 122 genes that were reported in the 15 most enriched GO biological process terms.

Supplementary Table S1 Descriptive statistics of dependent variables.

Supplementary Table S2 All lists of 111 categories from PANTHER Pathway analysis with significant genes detected by the differential expression analysis.

Supplementary Table S3 Top 20 GO Terms for all modules detected by the WGCNA.

Supplementary Table S4 List of upstream regulators identified by the Ingenuity's Upstream regulator Analysis.

Supplementary Table S5 The overall summary of alternative splicing events and results of Exact tests.

Supplementary Table S6 List of 122 genes that were identified in the 15 most enriched GO biological process terms (See the Table 1).

Supplementary Table S7 Results of the best subset regression model analyses.

Supplementary Table S8 Lists of Literatures for identified genes.
