# Supplementary figures and images for: Divergence in Morris Water Maze-Based Cognitive Performance under Chronic Stress Is Associated with the Hippocampal Whole Transcriptomic Modification in Mice
Source: Front Mol Neurosci. 2017 Aug 30;10:275. doi: 10.3389/fnmol.2017.00275 (PMC5582454; doi:10.3389/fnmol.2017.00275)

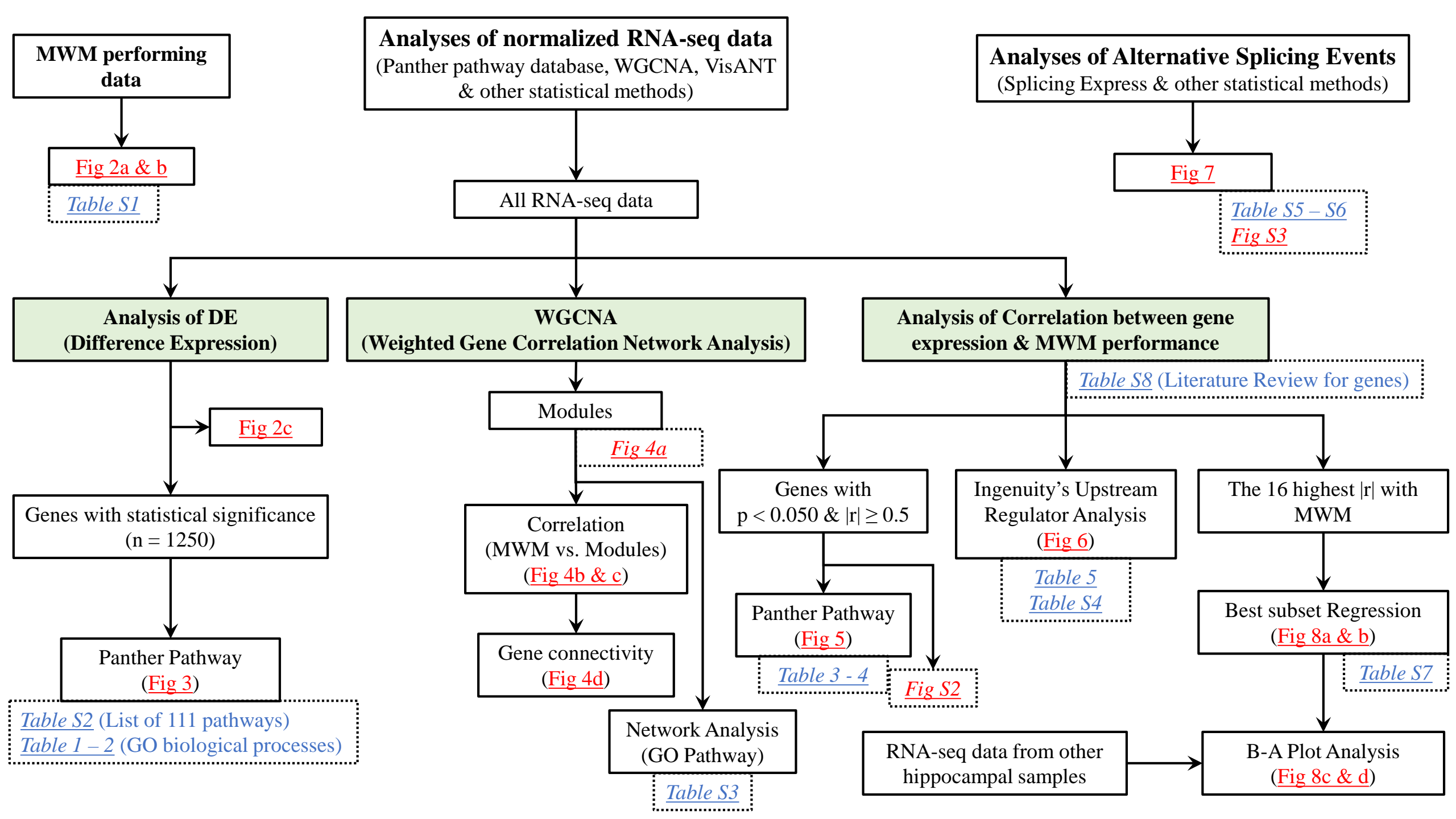

Supplement: Supplementary Figure S1 — The brief summary of data analysis methods for all tables and figures reported in the manuscript. [file Image1.PDF]

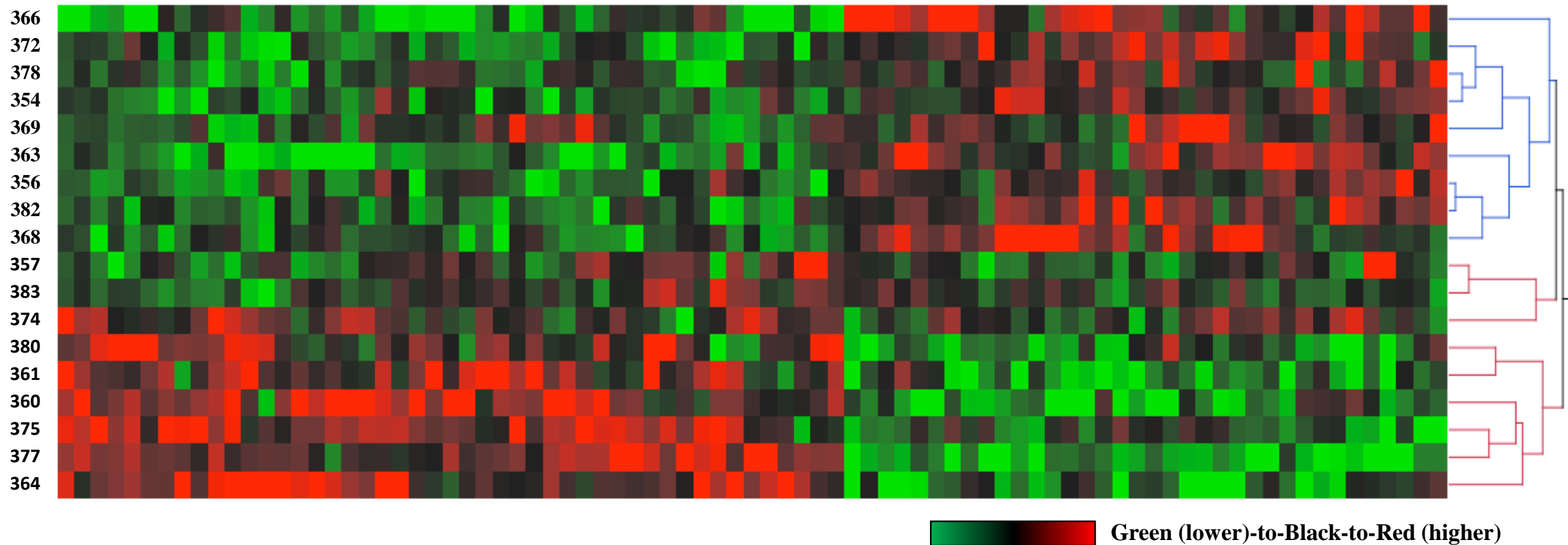

Supplement: Supplementary Figure S2 — Hierarchical Cluster with Dendrogram (Method = Ward) for significantly correlated genes with MWM1−5days Performance (# of genes = 82, p ≤ 0.010). [file Image2.PDF]

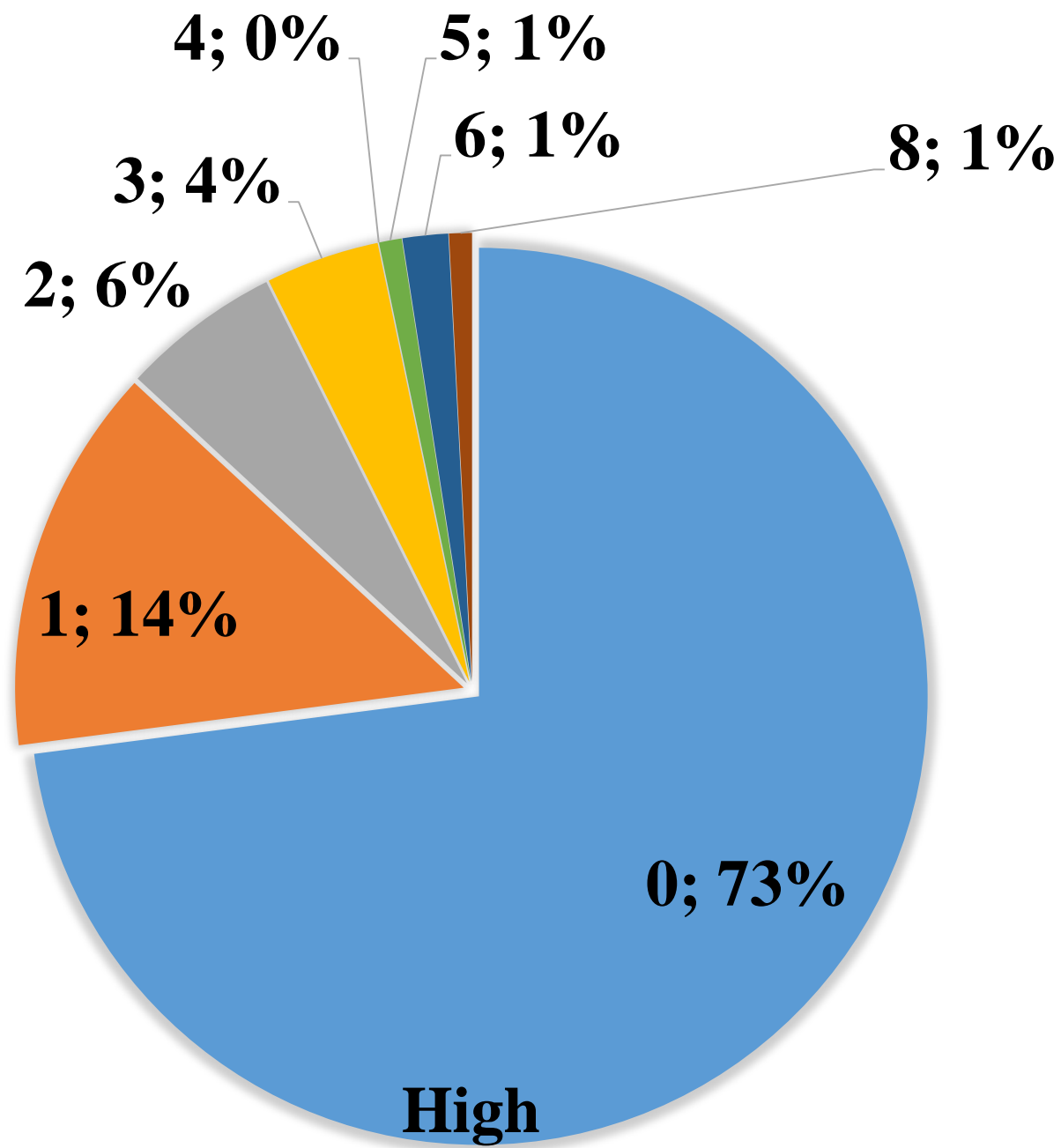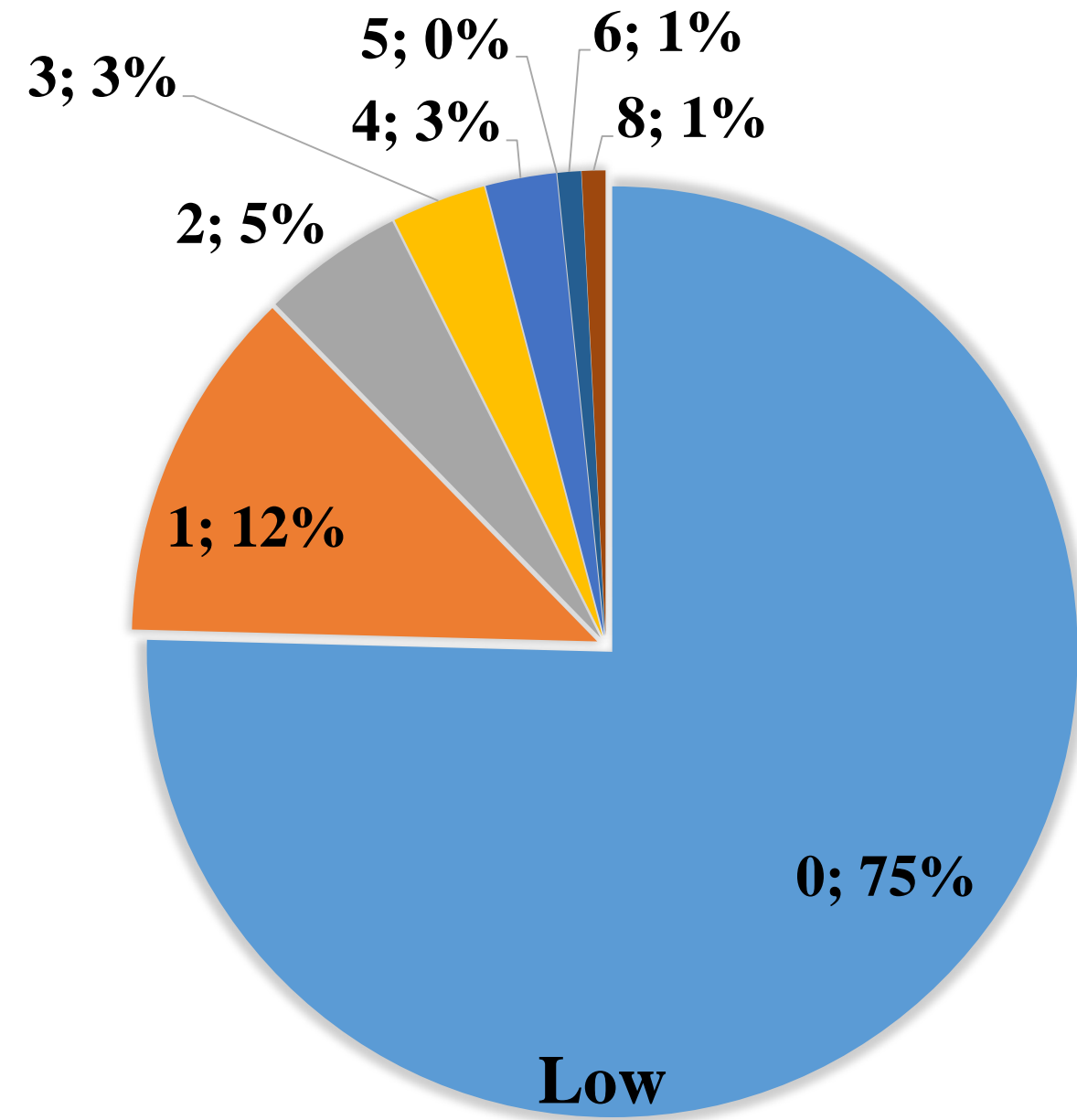

**MWM performance groups**

Supplement: Supplementary Figure S3 — Results of alternative splicing event types in 122 genes that were reported in the 15 most enriched GO biological process terms. [file Image3.PDF]
